# tReasure: R-based GUI package analyzing tRNA expression profiles from small RNA sequencing data

**DOI:** 10.1186/s12859-022-04691-1

**Published:** 2022-05-02

**Authors:** Jin-Ok Lee, Jiyon Chu, Gyuyeon Jang, Minho Lee, Yeun-Jun Chung

**Affiliations:** 1 Department of Biomedicine and Health Sciences, Graduate School, The Catholic University of Korea, Seoul, 06591 Republic of Korea; 2 Department of Life Science, Dongguk University-Seoul, Goyang, 10326 Republic of Korea; 3 Department of Microbiology, College of Medicine, The Catholic University of Korea, Seoul, 06591 Republic of Korea; 4 Precision Medicine Research Center, IRCGP, College of Medicine, The Catholic University of Korea, Seoul, 06591 Republic of Korea

**Keywords:** tRNA expression analysis, Small RNA sequence, GUI R packages

## Abstract

**Background:**

Recent deep sequencing technologies have proven to be valuable resources to gain insights into the expression profiles of diverse tRNAs. However, despite these technologies, the association of tRNAs with diverse diseases has not been explored in depth because analytical tools are lacking.

**Results:**

We developed a user-friendly tool, tRNA Expression Analysis Software Utilizing R for Easy use (tReasure), to analyze differentially expressed tRNAs (DEtRNAs) from deep sequencing data of small RNAs using R packages. tReasure can quantify individual mature tRNAs, isodecoders, and isoacceptors. By adopting stringent mapping strategies, tReasure supports the precise measurement of mature tRNA read counts. The whole analysis workflow for determining DEtRNAs (uploading FASTQ files, removing adapter sequences and poor-quality reads, mapping and quantifying tRNAs, filtering out low count tRNAs, determining DEtRNAs, and visualizing statistical analysis) can be performed with the tReasure package.

**Conclusions:**

tReasure is an open-source software available for download at https://treasure.pmrc.re.kr and will be indispensable for users who have little experience with command-line software to explore the biological implication of tRNA expression.

**Supplementary Information:**

The online version contains supplementary material available at 10.1186/s12859-022-04691-1.

## Background

Transfer RNAs (tRNAs) are 73–90 nucleotides (nt) long and are the most abundant small molecules that play a central role in protein synthesis [1]. tRNAs participate in diverse biological processes that maintain cellular vitality, such as cell proliferation, stress signaling, and apoptosis [2–4]. Thus, the dysfunction of tRNAs can affect diverse diseases, including cancer [1]. Regulation of tRNA expression is based on the translational requirement of cells, which reflects the difference in codon usage of protein-coding genes under different conditions [2, 5]. tRNA gene expression can influence the abundance of tRNA-derived fragments (tRF) [6]. Indeed, altered expression of tRNAs and tRF is involved in uncontrolled tumor cell growth, progression, and metastasis [7]. Isoacceptors are tRNAs carrying the same amino acids but expressing different anticodon sequences. Isodecoders are tRNAs bearing the same amino acids and anticodons but with sequence variations in the tRNA body [8]. More than 600 tRNA genes have been identified in humans, including 374 for isodecoders and 54 for isoacceptors [9]. Recent deep sequencing technologies have been valuable resources to gain insights into the expression profiles of diverse tRNAs [10, 11]. Moreover, a genomic tRNA database (GtRNAdb) that was recently developed and has become the most commonly used resource for tRNA information [9]. However, despite the availability of rich tRNA data resources, the contribution of various tRNAs to diverse diseases has not been well-studied owing to a lack of analytical tools. Although several tools, such as MINTmap [8] and sRNAbench [12] have been developed to analyze tRNA expression levels from small RNA sequencing data, they are still insufficient. In particular, none of the previous tools support predicting and quantifying mature tRNAs. Therefore, in this study, we aimed to develop a user-friendly analytical and visualization tool for studying differentially expressed tRNAs (DEtRNAs) associated with diseases. Unlike previous software packages, our new tool uses more stringent mapping strategies to better sort and quantify mature tRNAs from premature and non tRNAs.

## Implementation

**Fig. 1:**
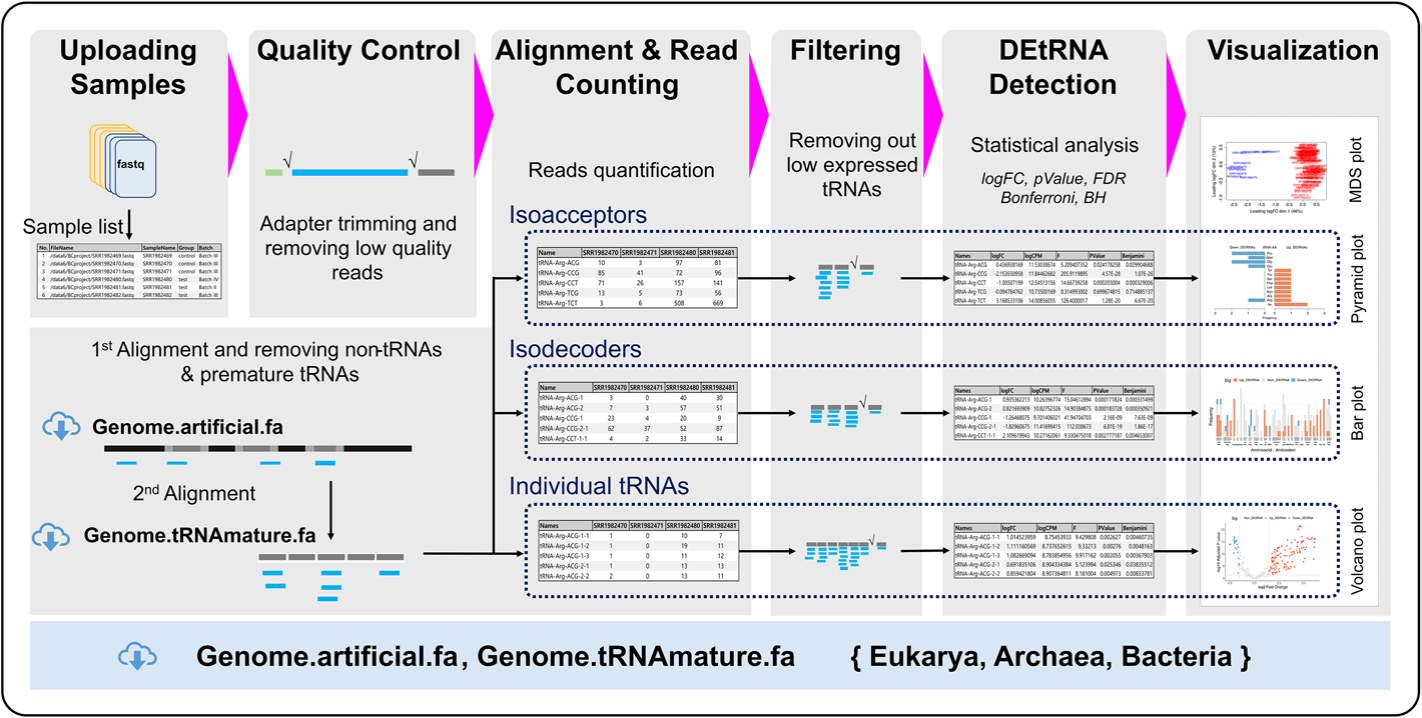
Overview of tReasure workflow

tReasure (tRNA Expression Analysis Software Utilizing R for Easy use) is a graphical user interface (GUI) tool for the analysis of tRNA expression profiles from deep-sequencing data of small RNAs (small RNA-seq) using R packages. The whole analysis workflow, including the uploading of FASTQ files of small RNA-seq, quantification of tRNA, defining DEtRNAs, and visualization of the statistical analysis, can be performed using the tReasure package (Fig. 1). tReasure requires an R computing environment and a gWidgets2 graphical library. Details of the installation and additional software packages for the analysis of small RNA-seq data are described in the user manual (Additional file 1). There are seven tabs in the main window. Each tab corresponds to a specific step in the workflow. Users can set the parameters, upload a dataset for analysis, detect DEtRNAs, perform statistical analysis, and visualize the analyzed results using tReasure.

### Sample uploading and quality control

Users can select FASTQ files for analysis from the directory of small RNA-seq files in the “Uploading samples” tab. tReasure supports only the FASTQ format with single-end reads as input files. To analyze tRNA expression profiles, adapter sequences and poor-quality reads were removed. tReasure supports this in the “Quality Control” tab by using the ShortRead package [13] and the “preprocessReads” function from the QuasR package [14]. Users can select the adaptor type, and then filter out the adapter sequences and poor-quality reads using user-defined parameters. tReasure provides four adapter types (Illumina smallRNA 3′ adapter, Illumina universal adapter, SOLiD adapter, and no adapter), two options of minimum quality threshold values (25 and 30), and a minimum length threshold. After running quality control, users obtain the total number of reads, number of reads that matched to the 3′ adapters, number of reads that were too small to analyze, and the number of reads that passed the filtering step. Details are available in the user manual (Additional file 1).

### Mapping and quantification of tRNA genes

#### Building an artificial tRNA genome

**Fig. 2:**
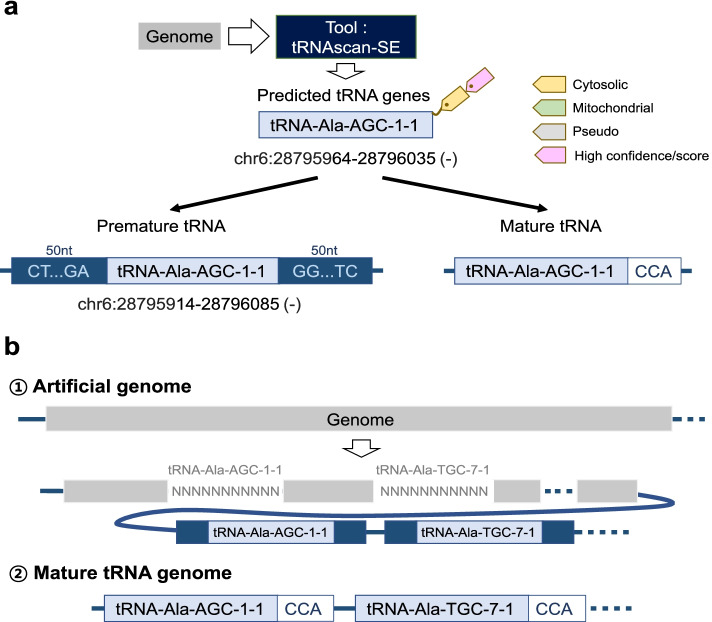
Building artificial tRNA genome and mature tRNA genome. **a** Generating premature and mature tRNA libraries from predicted tRNA genes. **b** Construction of the artificial genome and mature tRNA genome. Gray NNNNNNN represents masking annotated tRNA gene in the genome

Whole-genome sequence information for 4,781 species was downloaded from GtRNAdb. tRNAs were annotated using tRNAscan-SE [15] and GtRNAdb [9]. Using the predicted tRNAs, artificial and mature tRNA genomes were generated for each species (Fig. 2). tRNAscan-SE, one of the commonly used tools to predict tRNA genes, provides a score assigned to each putative tRNA gene. The genes with high scores are likely bona fide tRNA genes, while those with a low score are likely pseudogenes. tReasure uses tRNAs with high confidence scores that most likely function in translation by assessing a combination of domain-specific, isotype-specific, and secondary structure scores. To build the artificial genome, premature and mature tRNA libraries were generated using the predicted tRNA genes (Fig. 2a). Each of the premature tRNAs comprises the predicted tRNA sequence and 50 nt 3′ and 5′ flanking sequences, and each of the mature tRNAs was generated by appending 3′ CCA tails to the predicted tRNA sequence (Fig. 2a). An artificial genome was generated by masking all annotated tRNA genes and appending the premature tRNA library as additional chromosomes to the tRNA-masked genome (Fig. 2b). The mature tRNA genome was generated using the predicted mature tRNA library (Fig. 2b). Artificial and mature tRNA genomes of 4781 species (540 eukarya, 4024 bacteria, and 217 archaea) were generated. By selecting a species in the “Alignment & read counting” tab, tReasure provides the corresponding artificial genome from the tReasure web server.

#### Mapping mature tRNAs

**Fig. 3:**
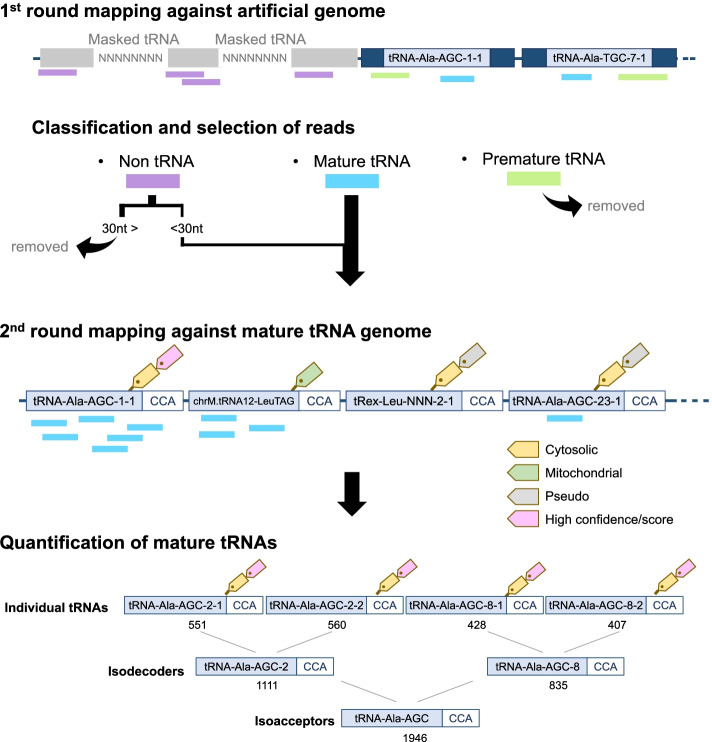
Mapping and quantification of mature tRNAs. The reads that mapped to the tRNA-masked genomic region are classified as “non-tRNA” (purple bars). The reads that mapped to the premature tRNA region of the additional chromosome are classified as “premature tRNA” (blue bars). The reads that mapped to the pure tRNA gene sequence are classified as “mature tRNA” (green bars). Only cytosolic and highly confident tRNAs are used for the final quantification of mature tRNAs. The numbers below each individual tRNA, isodecoder, and isoacceptor represent the mapped read counts

tReasure provides a two-step mapping process for specific mapping of mature tRNA genes based on a modification of a previous method [16] (Fig. 3). First-round mapping was used to remove the non-tRNA and premature tRNA reads. Second-round mapping was used to detect mature tRNA genes.

1. *First-round mapping against the artificial genome* Preprocessed small RNA-seq reads were aligned against the artificial genome using Rbowtie aligner (-v 3 --best, allowing up to three mismatches and selecting one optimal strand) based on the “qAlign” function of the QuasR package [14]. The reads are classified into three types based on mapping location in the artificial genome: (1) reads that mapped to the tRNA-masked genomic region were classified as “non-tRNA”; (2) reads that mapped to the premature tRNA region of the additional chromosome (tRNA sequence with 50 nt flanking sequences) were classified as “premature tRNA”; and (3) reads that mapped to the pure tRNA gene sequence (without the flanking sequences) of the additional chromosome were classified as “mature tRNA”. Of the three types of reads, mature tRNAs were used for second-round mapping. In addition, < 30 nt non-tRNA reads were also used for the second round, as described previously [16, 17] (Fig. 3). This process was performed using a customized R code.
2. *Second-round mapping against mature tRNA genome* For final mapping of the mature tRNAs, the sequence reads from the first-round mapping were aligned against the mature tRNA genome using Rbowtie aligner (-v 3 --best, same as the first mapping parameters). The reads that mapped to the mature tRNA genome were defined as mature tRNAs.

#### Quantification of mature tRNAs

For reliable and accurate quantification of mature tRNAs, tReasure uses only cytosolic tRNAs and tRNAs with high confidence values rather than the whole set of predicted mature tRNAs. The number of reads mapped to the individual tRNAs (cytosolic and high-confidence tRNAs) was counted using the Rsamtools package [18] (Fig. 3). tReasure can measure three different levels of mature tRNAs: individual tRNA, isodecoders, and isoacceptors. Isodecoders and isoacceptors were quantified by merging the counts of individual tRNAs using a customized R code (Fig. 3). After quantification, the count matrices generated per tRNA gene are displayed on each corresponding tab.

### Filtering out the tRNAs with low read counts

tReasure filters out tRNA genes with low read counts using the filtering tab. The counts per gene are normalized to counts per million (CPM) using the “cpm” function of the edgeR package [19].

### Identification of differentially expressed tRNA genes

To detect DEtRNAs between two groups, tReasure provides three statistical methods based on DESeq2 [20], EdgeR [19] and limma [21] packages using the “Detection of differentially expressed tRNAs” tab. These packages utilize “Relative Log Expression”, “Trimmed Mean of M-value (TMM)” or “Quantile” for normalization. For statistical analysis, EdgeR implements likelihood ratio tests, quasi-likelihood F-tests, and exact tests, while DEseq2 implements the Walt test. Limma implements the empirical Bayes statistical test method. tReasure provides multiple correction options in the form of false discovery rate, Bonferroni correction, and Benjamini–Hochberg (BH) methods. The output data contains the values of log fold change, log-CPM, F-statistic, raw p-value, and three types of adjusted p-values. All files are saved as tab-delimited texts.

### Visualization

tReasure can represent the results as multidimensional scaling (MDS), volcano, bar, and pyramid plots using the “Visualization” tab. The MDS plot shows the relative similarities between the samples based on the normalized expression data. The volcano plot shows the statistical significance and magnitude of the differences for individual tRNA genes. The bar plot represents the frequency of significantly up/downregulated tRNA-anticodons using the results of isodecoders. The pyramid plot represents the frequency of significantly up/down-regulated tRNA-amino acids using the results of the isoacceptors. tReasure provides the R code for customizing plots, such as font size, theme, and color. Details are available in the user manual (Additional file 1).

## Results

**Fig. 4:**
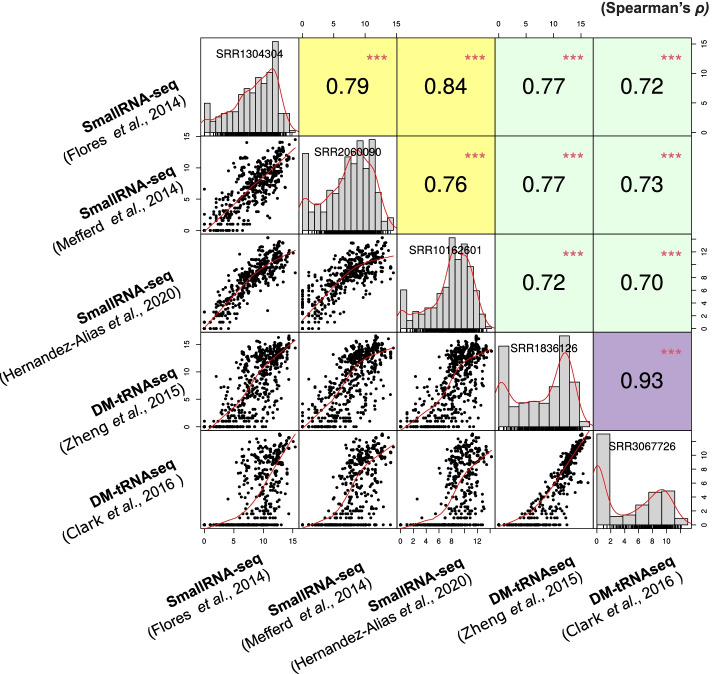
Spearman correlations between HEK293 cell tRNA quantifications. The graphs show Spearman correlations between HEK293 cell tRNA quantifications, including three small RNA-seq datasets and two well-established tRNA-seq datasets using DM-tRNA-seq. The scatter plots were log2-normalized for better visualization. Yellow boxes represent the coefficient of Spearman correlations between Small RNA-seq and Small RNA-seq. Green boxes represent the coefficient of Spearman correlations between Small RNA-seq and DM-tRNA-seq. Purple boxes represent the coefficient of Spearman correlations between DM-tRNA-seq and DM-tRNA-seq

To validate the mapping and quantification of tRNAs, we compared tRNA gene expression levels in HEK293 cells with five different datasets using tReasure: two datasets with demethylaze-tRNA-seq (DM-tRNA-seq) [22, 23] and three datasets obtained by small RNA-seq [17, 24, 25]. We performed an all-against-all pairwise correlation analysis of tRNA expression levels among the five datasets. The Spearman correlation coefficients between small RNA-seq and DM-tRNA-seq datasets ranged from 0.70 to 0.77, while the Spearman correlation coefficients across all samples ranged from 0.70 to 0.93 (Fig. 4). Our results are largely consistent with the Spearman correlations reported in previous studies using DM-tRNA-seq or Hydro-tRNA-seq data (ranging from 0.63 to 0.90) [17, 26], suggesting that tReasure can provide reliable tRNA quantification.

### Case study: identification of differentially expressed tRNAs using small RNA-seq data for breast cancer

**Fig. 5:**
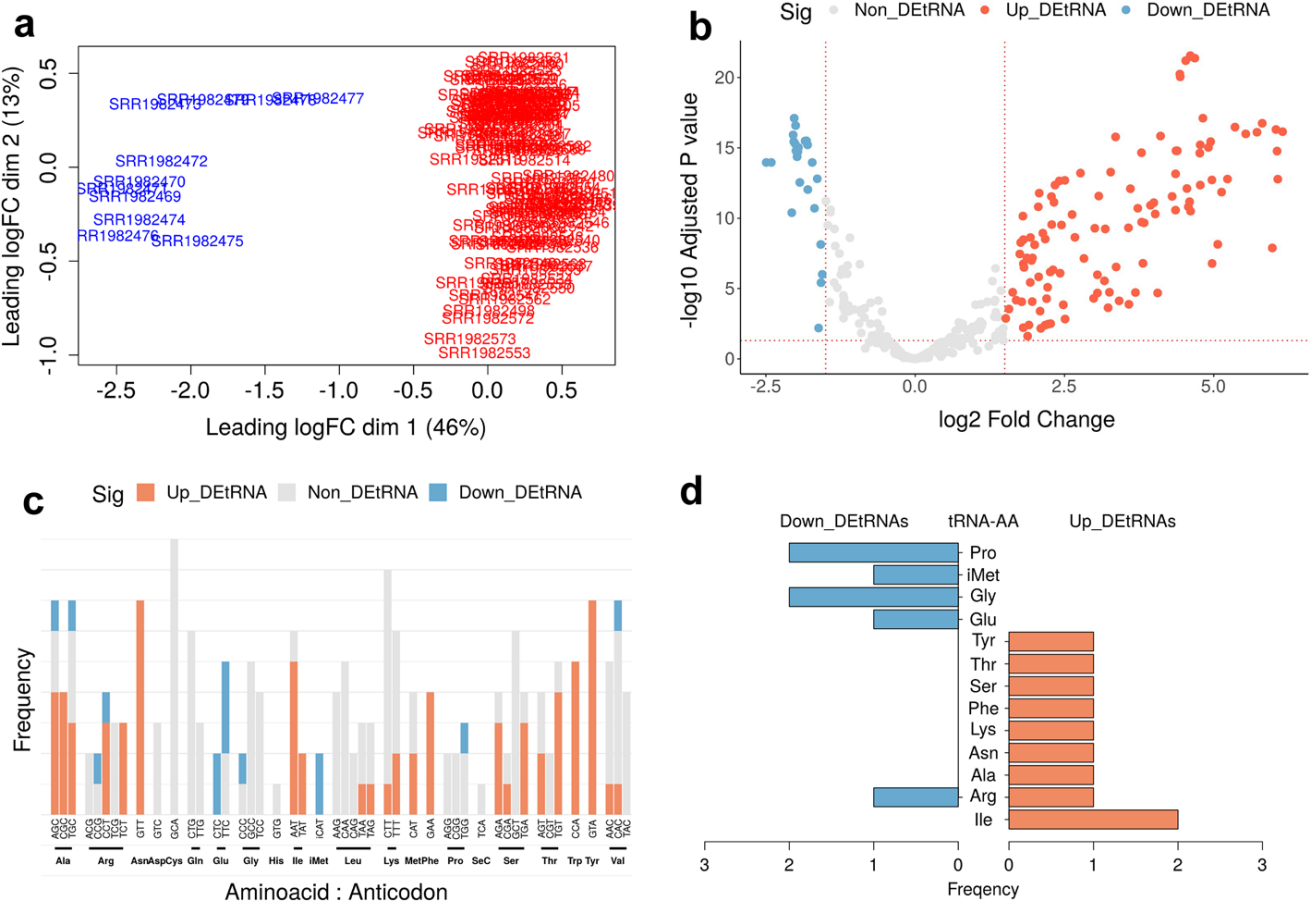
Plots of tReasure. **a** MDS plot. Healthy normal samples are the control group (blue) and breast cancer tumors are the test group (red). **b**–**d** Visualization of significantly differentially expressed genes between breast cancer and controls. Blue denotes downregulated tRNA gene expression, red denotes upregulated tRNA gene expression, and grey denotes no difference in expression. **b** Volcano plot of differentially expressed individual tRNAs. **c** Bar plot represents the frequency of significantly expressed tRNA-anticodons of isodecoders. **d** Pyramid plot displays the frequency of significantly expression of tRNA-amino acid of isoacceptors

We downloaded the FASTQ files of small RNA-seq data comprising 103 breast cancer tumors and 11 healthy normal breast tissues (GSE68085) [27]. The dataset contains Illumina smallRNA 3′ adapter sequences and four different batches of information. We set the parameters to remove adapter sequences (Illumina small RNA 3′ adapter) and trimmed the reads with Q-scores lower than 25. The preprocessed reads were aligned with the hg38 genome. Before the statistical analysis, tRNAs with a CPM value $\ge 1$ in at least 90% of the samples were retained for further analysis. All statistical analyses for the differential expression of tRNAs were performed using edgeR. Briefly, we normalized the data using the TMM method. When we checked the similarity or dissimilarity of the tRNA expression profiles between breast cancer and normal control groups using the MDS plot, all tRNAs of the breast cancer group were clustered on the right side, and all tRNAs of the control group were clustered on the left side without overlapping (Fig. 5a). For statistical significance, tRNA genes were filtered with a fold change > 1.5, and BH < 0.05. We identified 136 differentially expressed individual tRNAs (111 upregulated and 25 downregulated) in breast cancer tissue (Fig. 5b). Eighty-three isodecoders were differentially expressed (69 upregulated and 14 downregulated) between breast cancer and normal tissues (Fig. 5c). For example, the tRNA-Arg-CCG isodecoder family was downregulated, while the tRNA-Arg-TCT isodecoder family was upregulated in breast cancer tissue (Fig. 5c). Seventeen isoacceptors were differentially expressed (10 upregulated and 7 downregulated) (Fig. 5d).

## Discussion

In this study, we aimed to develop a user-friendly tool to analyze and visualize significant DEtRNAs associated with diseases. To reliably predict and quantify mature tRNAs, we developed several unique methods. Removing premature tRNA and non-tRNA reads before mapping is crucial to achieving precise quantification of mature tRNAs. We developed a unique step to obtain a more reliable mapping of mature tRNAs by modifying a previous method. In a previous study, to prepare a mature tRNA library as a template for quantifying tRNAs from the small RNA-seq data, predicted mature tRNAs were clustered based on the sequence identity of the tRNAs [16]. Therefore, mature tRNAs with identical sequences were clustered as one type of isodecoder, regardless of their chromosomal location in the mature tRNA library. However, to build the mature tRNA genome in tReasure, individual tRNAs were named and distinguished based not only on the sequence of tRNAs, but also on their chromosomal location. For example, the name “tRNA-Ala-AGC-2-1” represents the tRNA gene code for alanine, where the sequence classification is tRNA-Ala-AGC-2, and the genomic location is represented as the last digit (tRNA-Ala-AGC-2-1 and tRNA-Ala-AGC-2-2). In this way, tReasure can better quantify the individual mature tRNAs compared to previous attempts. tReasure also supports the quantification of isodecoders and isoacceptors by merging the read counts of individual tRNAs. For example, in the case of the breast cancer small RNA-seq data analysis (GSE68085) [27], read counts targeting tRNA-Ala-AGC-2-1 and tRNA-Ala-AGC-2-2 were 551 and 560, respectively; therefore, the read counts of tRNA-Ala-AGC-2 isodecoder were 1,111 (Fig. 3). However, the previous method could not distinguish individual tRNAs and isodecoders. To quantify the mature tRNAs, we selected only the tRNAs predicted to be cytosolic with a high confidence score as the template genome for small RNA-seq read mapping, rather than including all predicted tRNAs.

Several tools have been developed to analyze tRNA expression levels. MINTmap is a software package developed for the deterministic and exhaustive identification of tRFs in short RNA-seq datasets [8]. sRNAbench is a web-based tool to detect small RNA profiling and explore the differential expression of miRNAs, tRNAs, and rRNAs [12]. Although MINTmap supports the identification of tRFs, which are known to be involved in cancer tumorigenesis and progression, this tool is not user-friendly, especially for researchers unfamiliar with Linux. sRNAbench is a user-friendly tool; however, it is specialized for microRNA, so it is difficult to analyze DEtRNAs at the isodecoder and isoacceptor level with it, and it is not ideal for the quantification of mature tRNAs. Unlike previous tools, tReasure can quantify mature tRNAs and multiple levels of tRNAs (individual tRNAs, isodecoders, and isoacceptors) in addition to visualizing significant DEtRNAs associated with diseases.

A previous method [16] adopted realignment of the mapped reads to a mature tRNA reference using Segemehl [28], which allows up to 20 mismatches per 100 nt reads. tReasure does not require the realignment step and adopts a more stringent mapping condition (-v 3 --best, i.e., allowing up to three mismatches and selecting one optimal strand), enabling more precise measurement of read counts to tRNAs.

To verify the tRNA quantification performance of tReasure, we measured the tRNA expression levels and observed correlations among five different HEK293 cell datasets [17, 22–25] as described previously [17, 26]. Zhang et al validated their DM-tRNA-seq using HEK293 cell data, and showed a Spearman correlation of 0.73 between individual tRNAs expression levels [26]. Hernandez-Alias et al showed a Spearman correlation in the range of 0.63-0.9 in isodecoder expression levels using DM-tRNA-seq and Hydro-tRNA-seq [17]. Our results were largely consistent with the correlations observed in previous studies using DM-tRNA-seq or Hydro-tRNA-seq data [17, 26], suggesting that tReasure can provide reliable tRNA quantification. Although the biological implications and expression profiles of individual tRNAs associated with disease-associated DEtRNAs have not been studied yet in detail, tReasure might be helpful in exploring these factors in diverse diseases and species.

## Conclusion

Owing to the characteristics of tRNA structure, small RNA-seq data present fundamental limitations for detecting diverse tRNAs. These data can cover only a limited part of whole tRNAs and they tend to be biased toward tRF and unmodified tRNA. Although we developed several new methods for more reliable prediction and quantification of mature tRNAs in this study, we cannot exclude the possibility of tRFs interfering with small RNA-seq quantifications. Further studies are required to address this issue. tReasure is a user-friendly GUI package that supports the whole procedure of DEtRNA detection. The package uses FASTQ files of small RNA-seq data to visualize the DEtRNAs without additional analysis. It also supports the analysis of various species, from archaea to eukarya. We expect that tReasure will allow for exploring the biological implications of tRNA expression on an individual’s desktop computer.

## Availability and requirements

Project name : tReasure: R-based GUI package analyzing tRNA expression profiles from small RNA sequencing data. Project home page: https://treasure.pmrc.re.kr. Operating system(s): Window, Mac OS and Linux. Programming language : R License: GNU GPL 3.0 Any restrictions to use by non-academics : None

## Supplementary Information

**Additional file 1.** User manual. Detailed instructuons for using tReasure.

## Data Availability

The source code is available on at https://treasure.pmrc.re.kr.

## Abbreviations

BH: Benjamini–Hochberg
CPM: Counts per million
DE: Differentially expressed
DEtRNAs: Differentially expressed tRNAs
DMt-RNA-seq: Demethylaze-tRNA-seq
FC: Fold change
GtRNAdb: The genomic tRNA database
GUI: Graphic user interface
MDS: Multidimensional scaling
TMM: Trimmed Mean of M-value
tReasure: tRNA Expression Analysis Software Utilizing R for Easy use
tRF: tRNA-derived fragments
tRNA: Transfer RNA
QC: Quality Control
