# Supplementary material for: tReasure: R-based GUI package analyzing tRNA expression profiles from small RNA sequencing data
Source: BMC Bioinformatics. 2022 May 2;23:155. doi: 10.1186/s12859-022-04691-1 (PMC9063265; doi:10.1186/s12859-022-04691-1)
Supplement: Supplementary file 1 — Additional file 1. User manual. Detailed instructuons for using tReasure. [file 12859_2022_4691_MOESM1_ESM.pdf]

tReasure

(tRNA Expression Analysis Software Utilizing R for Easy use)

User Manual

Ver. 1.0.0

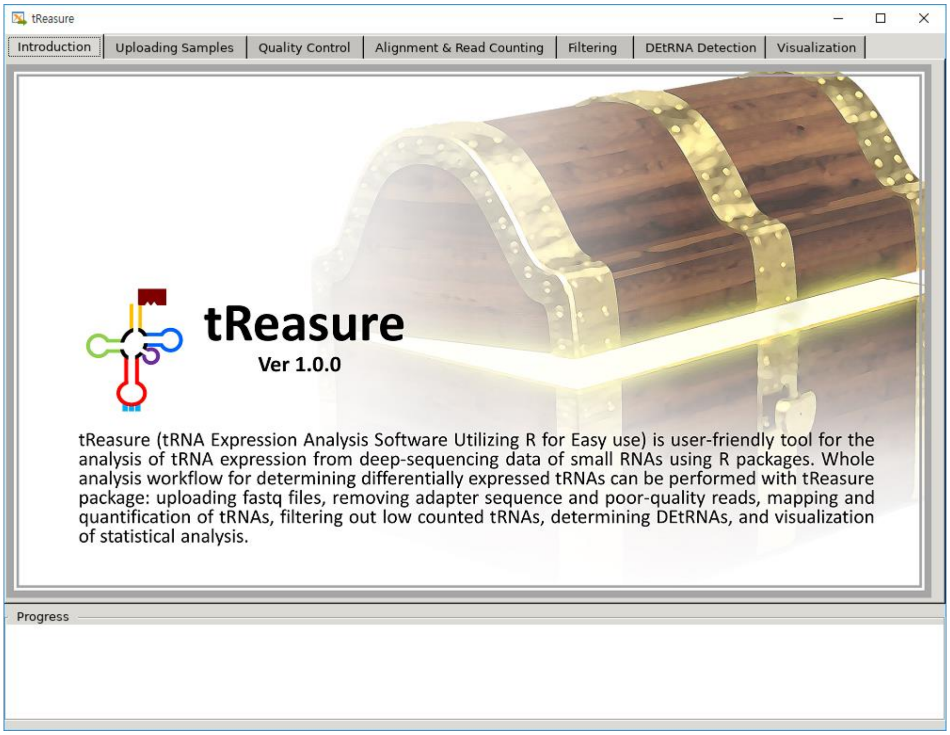

Contact: MinhoLee@dgu.edu or yejun@catholic.ac.kr

Table of Contents

1. Introduction 2

1.1. Installation 2

2. Start 3

3. User Interface 3

3.1. Tab “Uploading Samples” 4

3.1.1. How to make a sample list 4

3.1.2. Working directory 9

3.2. Tab “Quality Control” 9

3.2.1. Workflow of analysis 9

3.3. Tab “Alignment & Read Counting” 12

3.3.1. tRNA mapping strategy 12

3.3.2. Quantification of tRNAs 12

3.3.3. Workflow of alignment and read counting 13

3.4. Tab “Filtering” 17

3.4.1. Workflow of filtering 17

3.5. Tab “DEtRNA Detection” 19

3.5.1. Workflow of DEtRNA Detection 19

3.6. Tab “Visualization” 23

3.6.1. Customizing plots 26

4. Option 28

5. Reference 30

1. Introduction

tReasure (tRNA Expression Analysis Software Utilizing R for Easy use) is a user-friendly tool for the analysis of tRNA expression from deep-sequencing data of small RNAs using R packages. tReasure package includes several RNA-seq R packages, which are available in www.bioconductor.org. tReasure covers the whole analysis workflow of high throughput sequencing experiments to identify and visualize of differentially expressed tRNAs using FASTQ File format.

tReasure is a package for the R computing environment; therefore, you must install R and Rstudio (https://rstudio.com) before installing tReasure. tReasure requires the gwidget2 graphical library to run and several additional packages for the analysis of RNA-seq.

1.1. Installation

- **Preliminaries**

  - For Window: User need to install Rtools.

  - For Linux: Users need to install libcurl4-openssl-dev and libssl-dev for devtools and 'libgtk2.0-dev' and 'libxml2-dev' for tReasure.

    (Example for Ubuntu)

```
#installing for devtools
>sudo apt-get install libcurl4-openssl-dev
> sudo apt-get install libssl-dev

#installing for tReasure
>sudo apt-get update -y
>sudo apt-get install -y libxml2-dev
>sudo apt-get install -y libgtk2.0-dev
```

  - For Mac : Users need to install 'XQuartz', gtk2 and cairo.

    (Example using brew)

```
#installing for devtools
>brew update
>brew install --cask xquartz
>brew install openssl

#installing for tReasure
>brew update
>brew install cairo
>brew install gtk+
```

- **Installation for tReasure**

Open Rstudio or R and type as below:

```
>install.packages("devtools")
>library("devtools")

>devtools::install_github("jinoklee/tReasure",force = TRUE)
>library("tReasure")
>tReasure::install.tReasure()
```

If the description shows as below during the installation, choose **"Install GTK+".**

```
 Need GTK+? (Restart Required)
     Install GTK+
     Do not install GTK+
```

2. Running tReasure

➢ **Open Rstudio or R and type as below**

```
>library("tReasure")
>tReasure::tReasure()
```

3. User Interface

On the main page of tReasure, Tabs for analysis were indicated below; Introduction, Uploading Samples, Quality Control, Alignment & Counting, filtering, DetRNA Detection, Visualization. Each tab corresponds to a particular step of the analysis workflow. Left panel contains the user-defined parameters, Right panel represents the output of each process, and Bottom panel shows analysis progress (Figure 1).

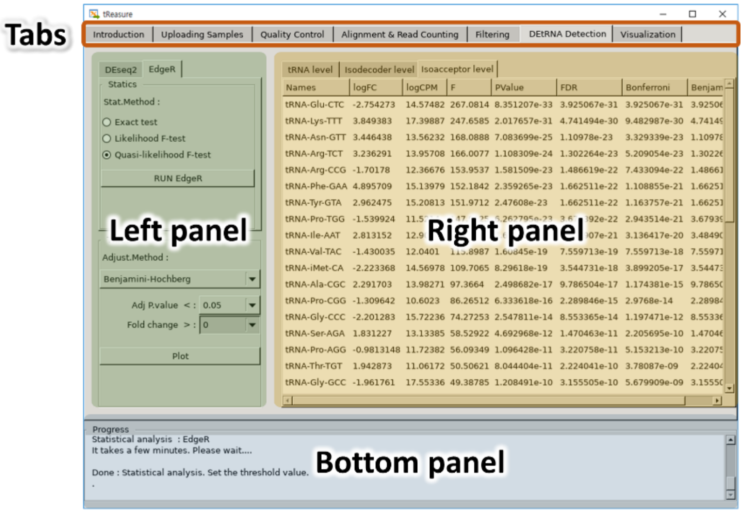

Figure 1. Main page of tReasure software User Interface

Above all, prepare small RNA-seq dataset formatted FASTQ. We provided an example dataset (smallRNA-seq data, GSE68085) [1] for practicing the analysis. The example dataset contains 114 breast tissues of small RNA-seq data which is the total of 103 tumors and 11 normal.

3.1. Tab "Uploading Samples"

Before starting analysis, create a folder (e.g., named "BCproject") on your local computer and move the dataset to your local folder. This folder will be set as a working directory and as a storage of outputs, later.

3.1.1. How to make a sample list

① Click "Open" button on the Left panel for selecting the directory of raw FASTQ files (Figure2).

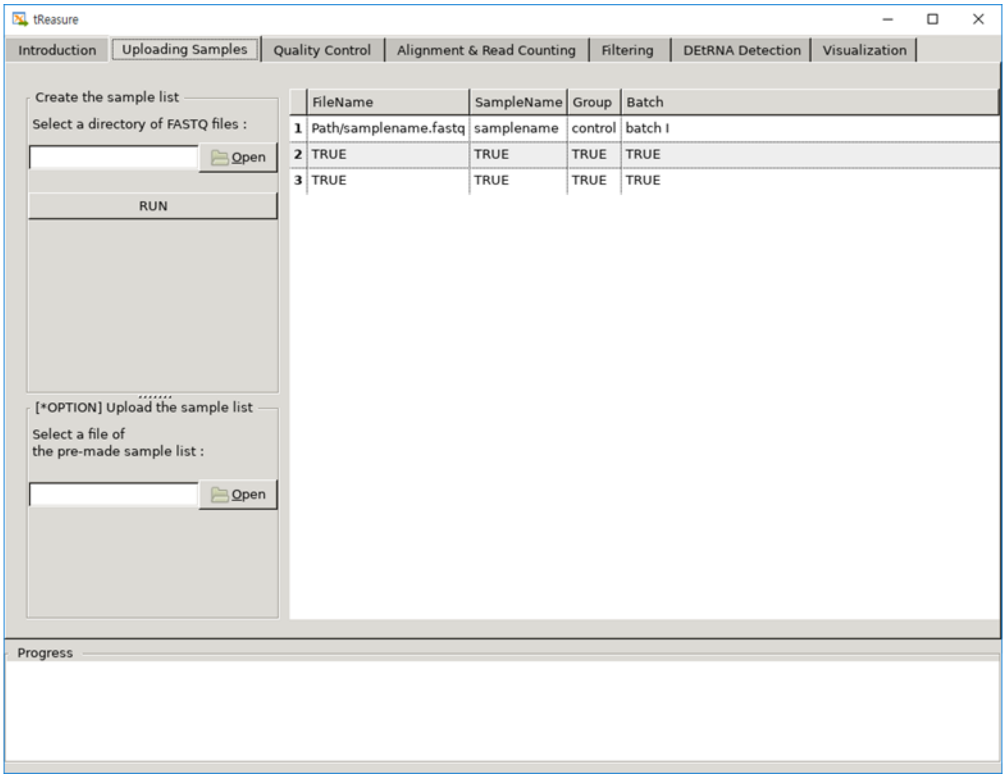

Figure 2. Tab of "**Uploading samples**"

⇨ A new pop-up window will be appeared. Left panel shows directories and Right panel shows the contents in the current directory. Top panel shows the current directory (Figure 3).

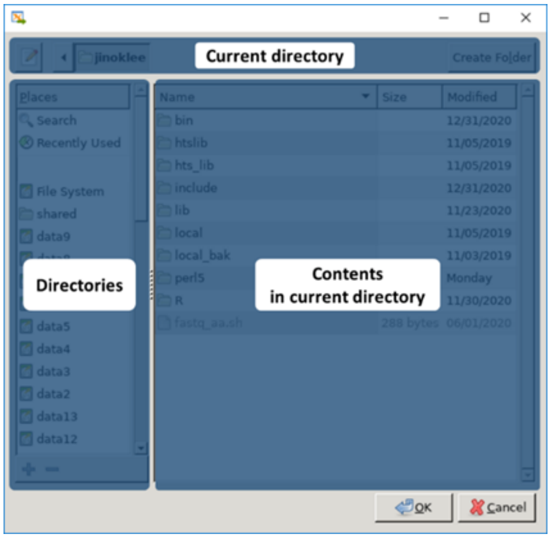

Figure 3. Pop-up window for selecting directory of raw FASTQ files

② Search your local folder (e.g., “BCproject”) by clicking the folders on Upper or Left panel of pop-up window (Figure 4).

⇨ The path of clicked folders is showing the top of pop-up window (e.g., /data6/BCproject).

③ Click “OK” button (Figure4).

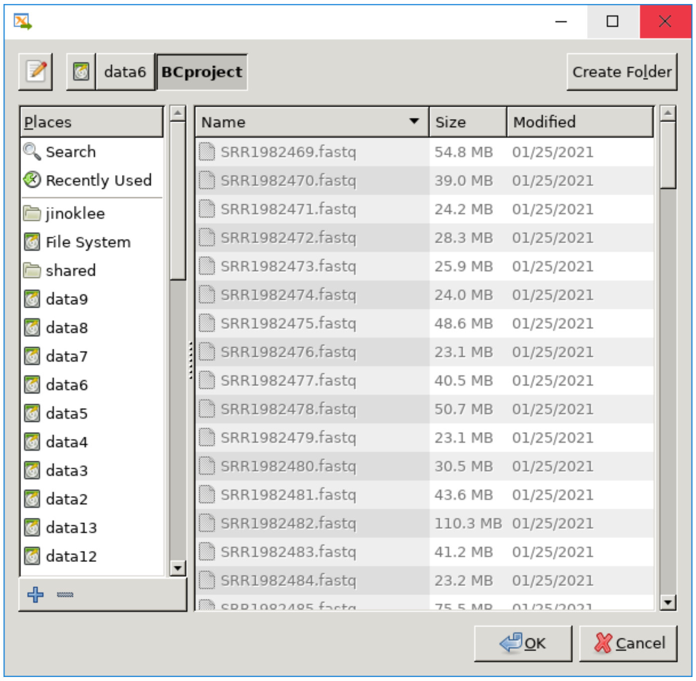

Figure 4. Searching your local folder

⇨ The FASTQ files in the folder is selected for the analysis

④ Click “RUN” button on the Left panel for making the sample list (Figure 5).

⇨ The sample list is displayed on the Right panel and the list is saved in a folder named as “sample.txt” at the same time (i.e., /data6/BCproject/sa mple.txt).

⇨ There are four columns named “FileName”, “SampleName”, “Group”, and “Batch” (Figure 5).

FileName: Paths and names of the files containing the raw data. The con tents generated automatically according to the information of files.

SampleName: The sample names of each sequence data and create fro m raw data filename. The contents generated automatically according t o the information of files.

Group: The group information of samples. There are filled with one of “c ontrol’, “test” or “NA” by default.

Batch: A batch information of samples. There are filled with "NA" by def ault.

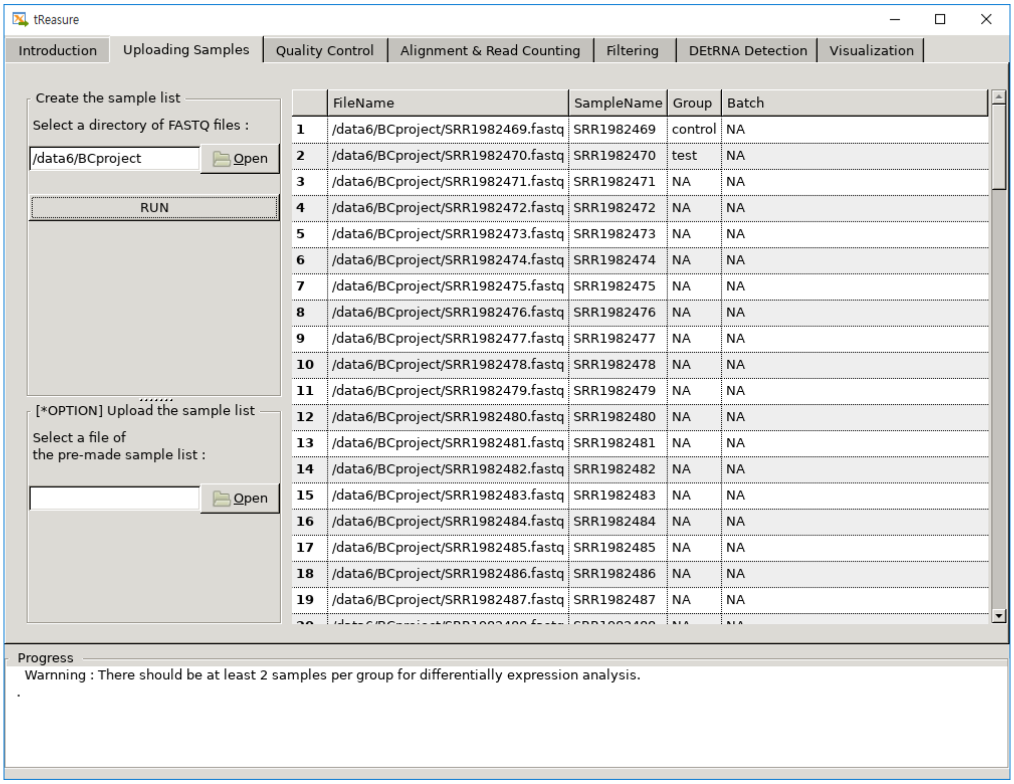

Figure 5. Creating sample list with default values

⑤ Modified the sample list.

Note. There are two methods to modify those.

First, you can directly select the group information and add the batch information on the Right panel. The revised sample list is automatically saved as “sample.txt” (Figure 6).

Second, you can modify a saved file (i.e., “sample.txt’ of ④) using Microsoft Excel. After modifying the sample list in Excel, you must save it as text format (tab delimited) without changing the file name and sample name. Then, click “Open” in the [*OPTION] box on the Left panel (Figure 7).

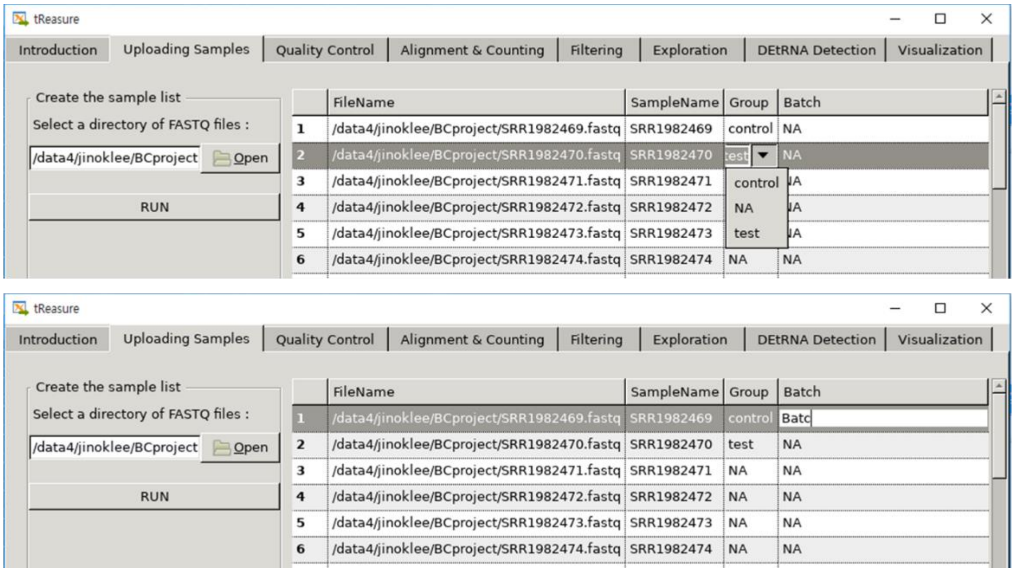

Figure 6. Directly modified sample list

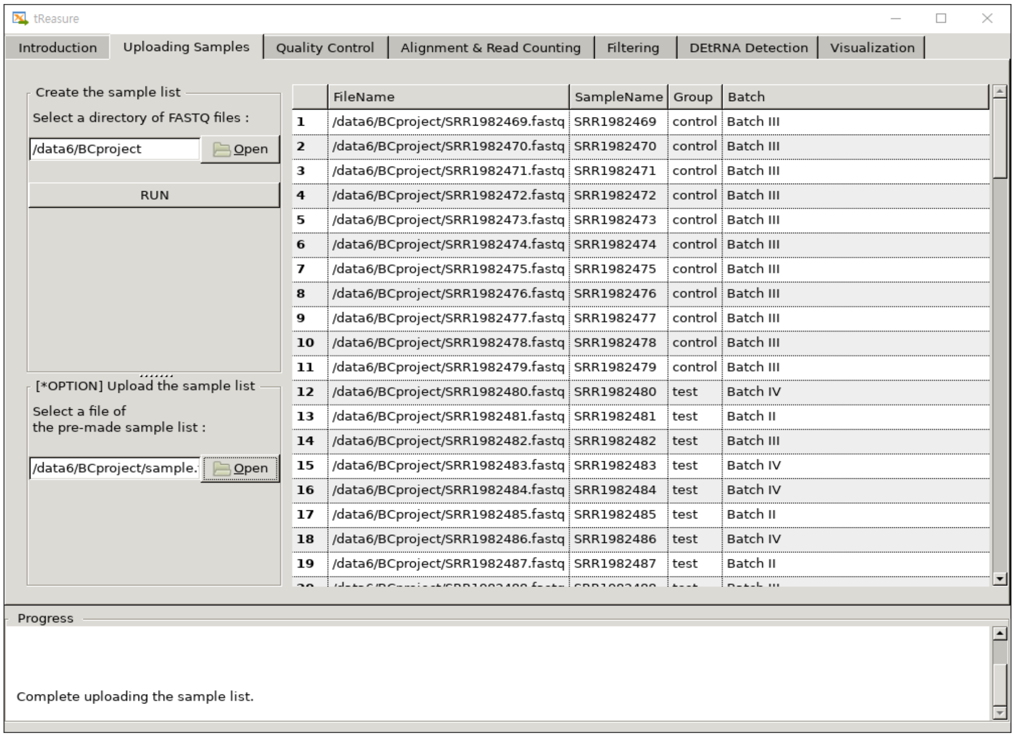

Figure 7. Example of making the sample list

Caution. There should be at least two replicates in one group for statistical analysis of differential gene expression.

Caution. Don't click the "RUN" button again after finishing modification. Move on to the next step ("**Quality Control**" tab). If user click "RUN" button after modification, it can reset the sample list.

3.1.2. Working directory

Once you click "RUN" (④) button for making sample list, the selected folder is set as the working directory ($WORKDIR) automatically (i.e., $WORKDIR = /data6/BCproject). At the same time, four subdirectories will be created to save the outputs of subsequence procedures such as $WORKDIR/pre, $WORKDIR/post, $WORKDIR/rc and $WORKDIR/stat/plot (Figure 8).

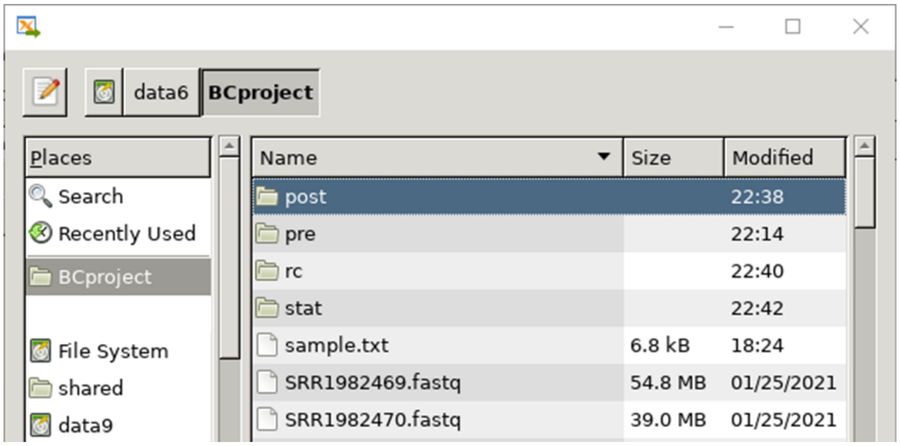

Figure 8. Setting the working directory and making subdirectories

3.2. Tab "Quality Control"

As a next step, you need to remove adapter sequence and poor-quality reads. Those are performed using *preprocessReads* function from the package *QuasR* [2].

3.2.1. Workflow of analysis

First, check the adapter information in small RNA-seq data. tReasure provides four options of adapters (Illumina smallRNA 3' adapter, Illumina universal adapter, SOLiD adapter, and No adapter) (Figure 9). Second, choose the threshold value of Q-score (quality score) to filter out low quality reads. tReasure provides two values ("25" and "30") for minimum quality threshold. Regarding minimum length, tReasure provides "10" as a threshold for optimal detection of tRNAs from small RNA-seq.

① Select the user-defined parameters on Left panel. Figure 9 is an example.

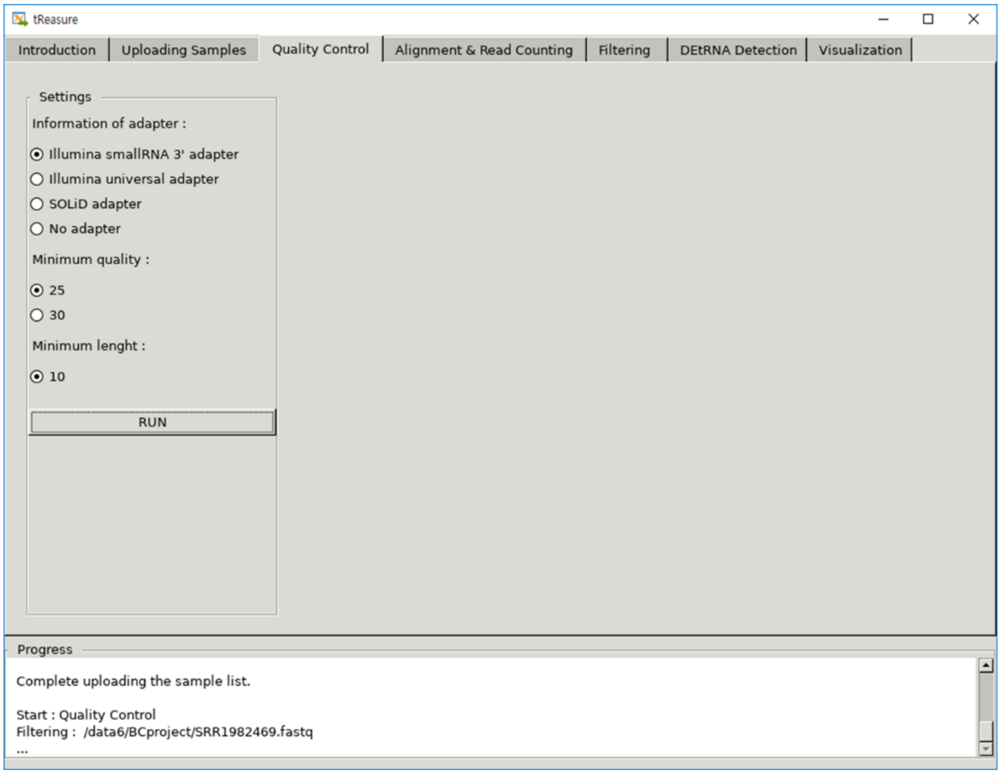

Figure 9. Tab of "**Quality Control**"

② Click "RUN".

⇨ The summary of Quality Control (QC) is displayed on the Right panel and saved in the subdirectory ("$WORKDIR/pre") named as "trim_res.txt (Figure 10-11).

⇨ Each column represents individual small RNA seq data, they have four kinds of QC information (totalSequences, matchTo3pAdapter, tooShort, and totalPassed).

totalSequences: total number of reads

matchTo3pAdapter: number of reads that matched to the 3' adapters

tooShort: number of reads that were too short to analyze

totalPassed: number of reads that passed the filtering step

⇨ Trimmed FASTQ files are saved in that directory "_trim.fastq" (Figure 11).

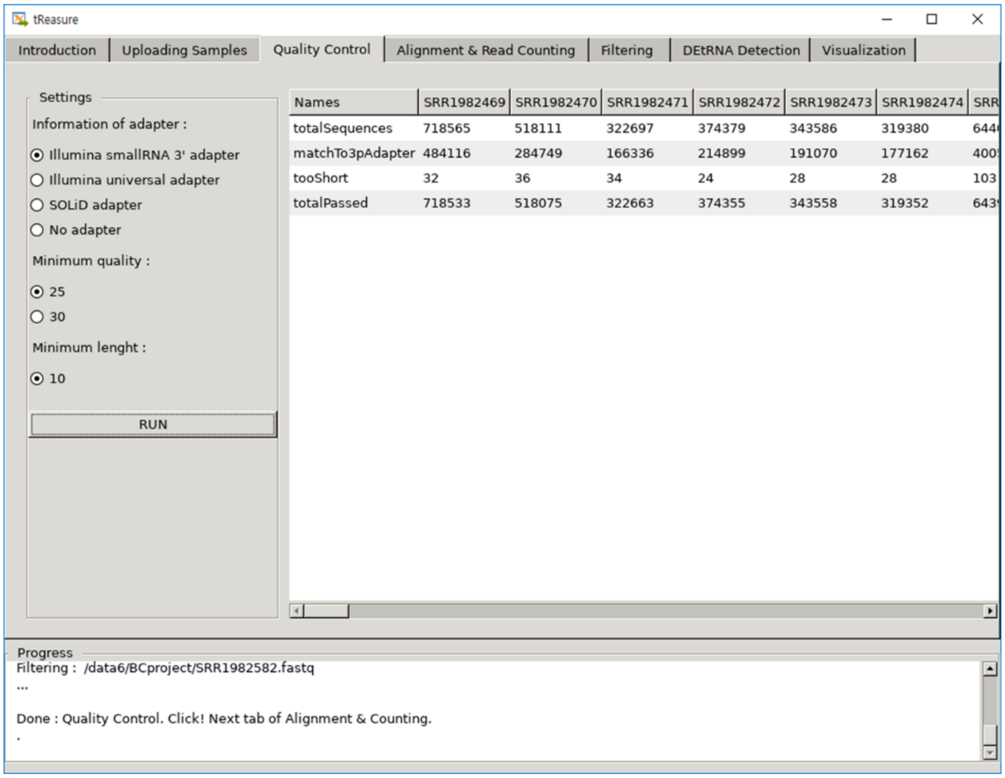

Figure 10. Summary of QC

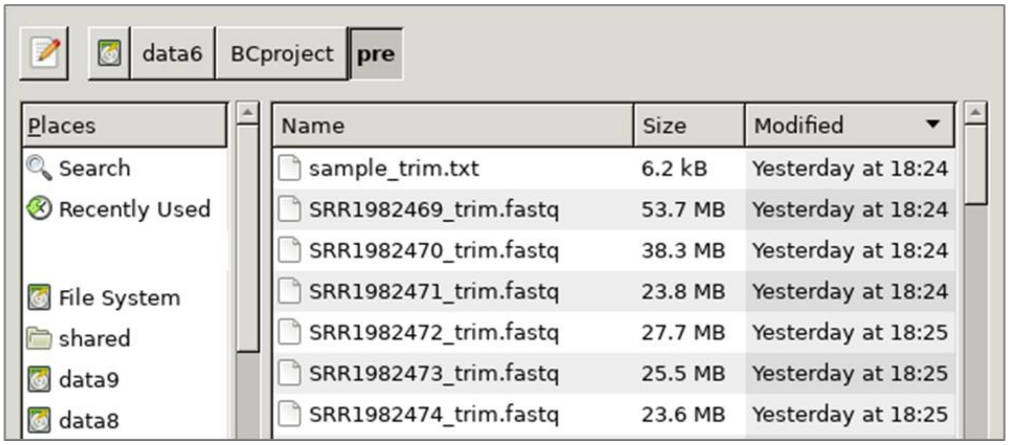

Figure 11. Output of QC

Note. The process of status is displayed on the Bottom panel.

3.3. Tab "Alignment & Read Counting"

After QC, tReasure provides read alignments against genome assembly using *Rbowtie* aligner based on *qAlign* function of *QuasR* packages [2]. *Rsamtools* [3] package is used for filtering and counting of reads.

3.3.1. tRNA mapping strategy

tReasure performs a specific mapping method for tRNA genes, which is modified a previous method [4]. In brief, an artificial genome is generated by masking all annotated tRNA genes and adding pre-tRNA genes (i.e., tRNA genes with 3' and 5' genomic flanking regions) as extra chromosomes. tRNA annotations are obtained by using tRNAscan-SE [5], which predicted all tRNA sequence. Upon mapping to this artificial genome by *Rbowtie*, sequence reads that map to the tRNA-masked chromosomes or to the tRNA flanking regions are filtered out to remove non-tRNA reads and unmatured-tRNA reads, respectively.

tRNAs without flanking region are transformed to mature tRNAs by appending 3' CCA tails and removing introns. The subset of filtered reads from the first mapping is aligned against the mature tRNAs using *Rbowtie*.

tReasure provides artificial genome and mature tRNAs sequence of 4,781 species (540 eukarya, 4,024 bacteria, and 217 archaea). When starting alignment, the genome files of your choice are automatically downloaded from tReasure webserver.

3.3.2. Quantification of tRNAs

tRNAscan-SE, a frequently used tool to predict tRNA genes, is provided a score assigned to each putative tRNA gene. The genes with high score (>50) are likely bona fide tRNA genes, while those ranked with a low score are likely pseudogenes [5]. And tRNAscan-SE determines the "high confidence" set of genes that are most likely function in the translation process, by assessing a combination of domain-specific, isotype-specific, and secondary structure scores. As a benchmark, tReasure counts and uses only the cytosolic and high confidence tRNAs of all predicted them.

The identity of each tRNA is defined by its corresponding amino acid and by its ant icodon sequence. tRNAs charged with the same amino acid are isoacceptor tRNAs (e.g., tRNA-Arg), and tRNAs with the same anticodon sequence are known as isod ecoder tRNAs (e.g., tRNA-Arg-TCT). In this work, tReasure combines tRNA genes h aving the same mature tRNA sequence into tRNA families. In other words, tReasur e quantify the mapped reads of isodecoder genes that a single isodecoder set wer e assigned to individual tRNA genes (e.g., tRNA-Arg-CCG-2-1), and multiple tRNA g enes with identical sequences were assigned to a single "tRNA family" (e.g., tRNA-

Arg-ACG-1) [6] (Figure 12). By using these concepts, tReasure is capable of detecting at a broad level from individual to isodecoder, isoacceptor.

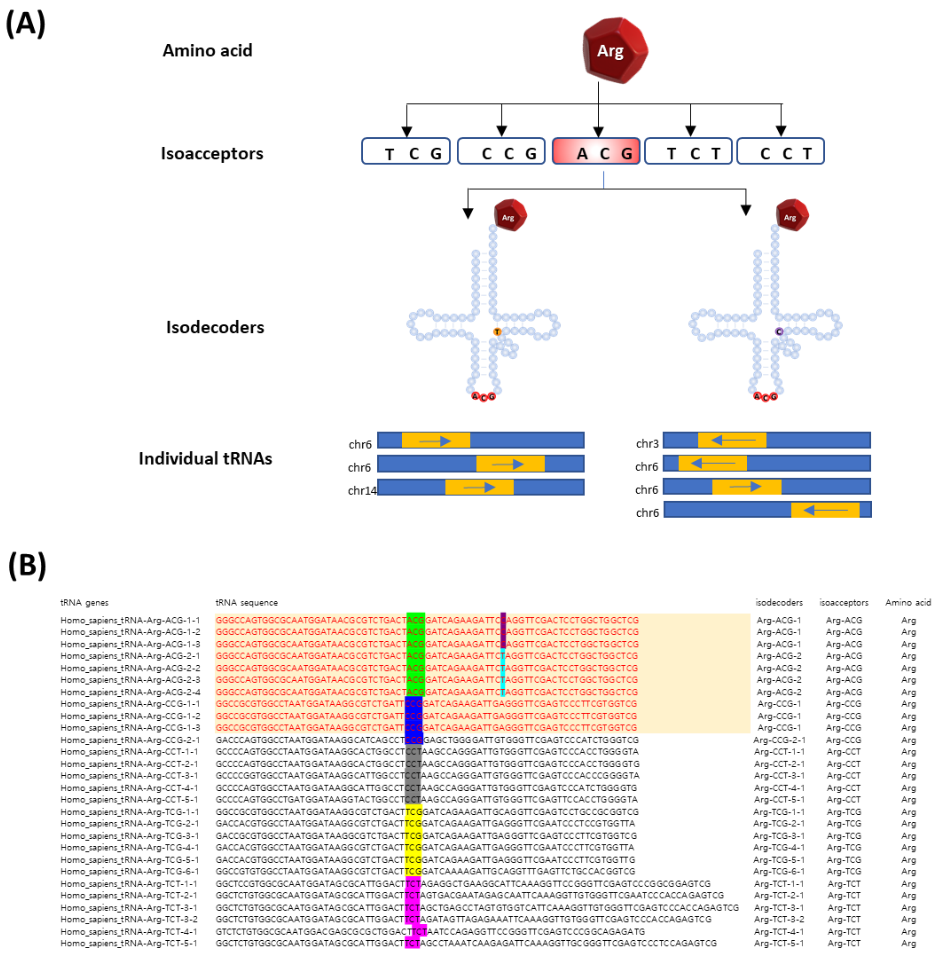

Figure 12. Definition of tRNAs

3.3.3. Workflow of alignment and read counting

First, select genome assembly according to your studies.

① Click the button named “Search genome assembly” on the Left panel (Figure 13).

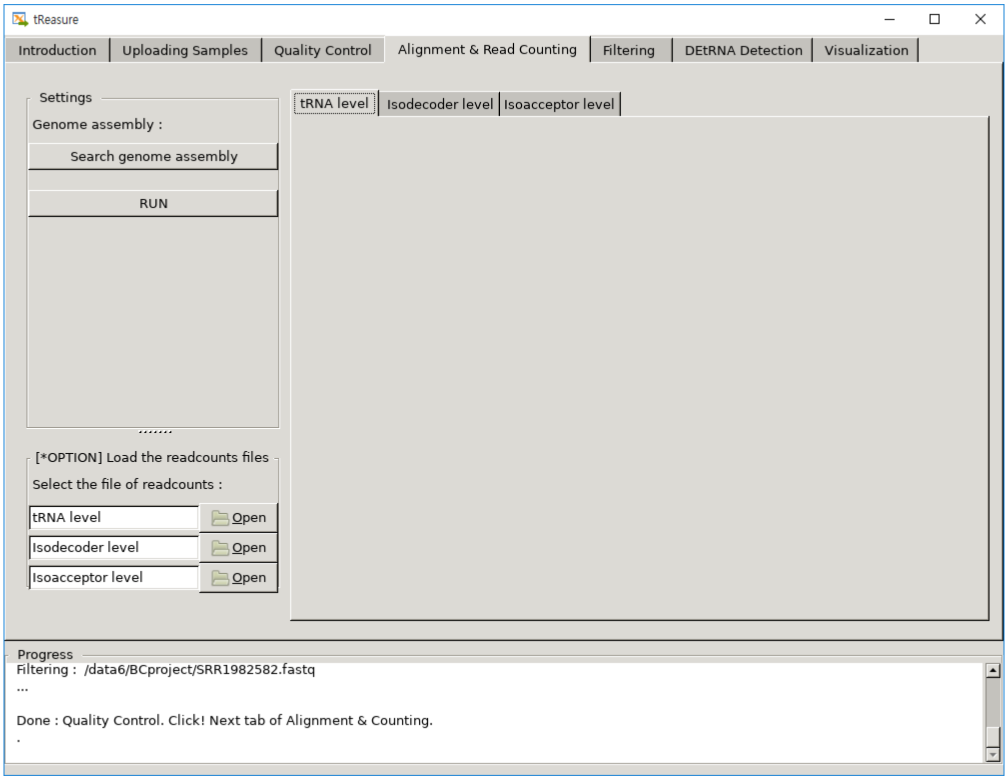

Figure 13. Tab of “**Alignment & Read Counting**”

⇨ The mini pop-up window is appearing for selecting the genome assembly you want (Figure 14).

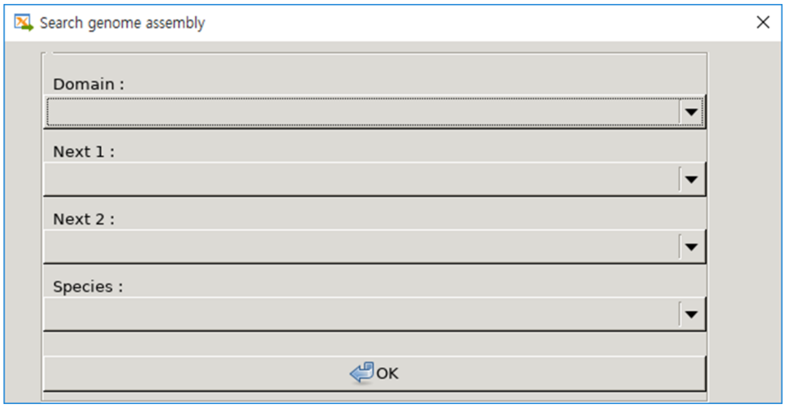

Figure 14. Mini pop-up window for searching genome

② Select the four criteria and click “OK” (Figure 15).

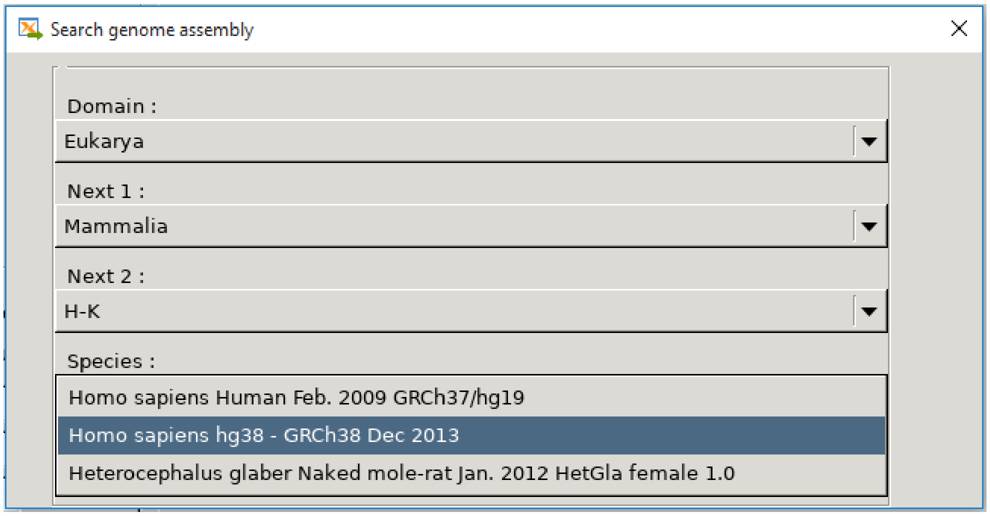

Figure 15. Example of selecting the gnome assembly

⇨ The mini pop-up window is closed, and the name of genome is displayed on Left panel of main window.

③ Click "RUN" button on the Left panel.

⇨ tReasure automatically download both artificial genome (e.g., Hsapi38_artificial.fa) and mature tRNA sequence (e.g., Hsapi38.tRNAscan_mature.fa) of selected species formatted zip file (Figure 16). Those are stored in the folder of the default library path (e.g.,C:/Users/[Username]/Documents/R/win_library/3.6/tReasure/extdata/refer/Hsapi38/).

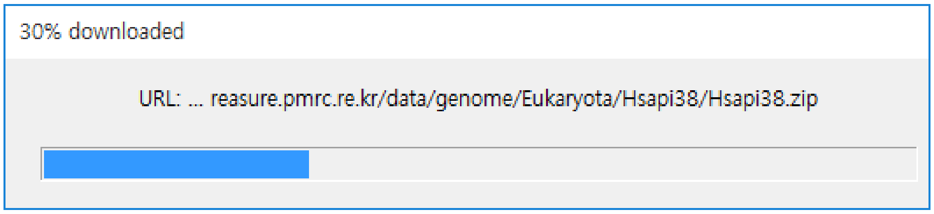

Figure 16. Downloading of files for alignment

Note. Before aligning the small RNA seq reads against a reference genome, it is necessary to do indexing on the genome. To reduce execution time, we provide the pre_built index of eukaryotic genome, which is also downloaded with genome.

⇨ tReasure goes through three steps automatically one by one: pre-mapping, postprocessing, and counting of reads.

After first pre-mapping step, the BAM files are saved in subdirectory ("$WORKDIR/pre"). And the summary of alignment is also saved named as

"preprocessing_algin_stat.txt", which contains the size of the target sequence as well as the number of mapped/unmapped reads for each sequence file (Figure 17).

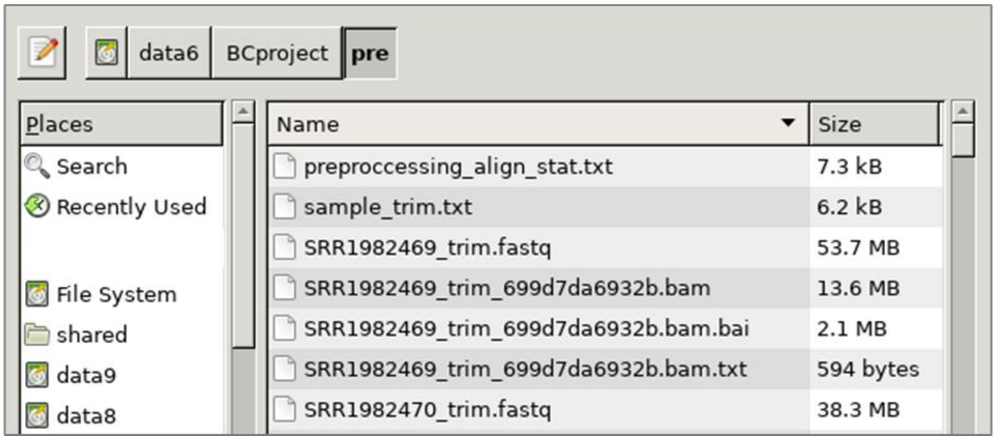

Figure 17. The output of pre-mapping step

After postprocessing step, results are saved in subdirectory ("$WORKDIR/post"). There are FASTQ files for the secondary library of mature tRNAs named "_mature" to the end of the filename, and the BAM files of outputs of mapping against mature tRNAs. Likewise, the summary of alignment is saved as "postprocessing_align_stat.txt" (Figure 18).

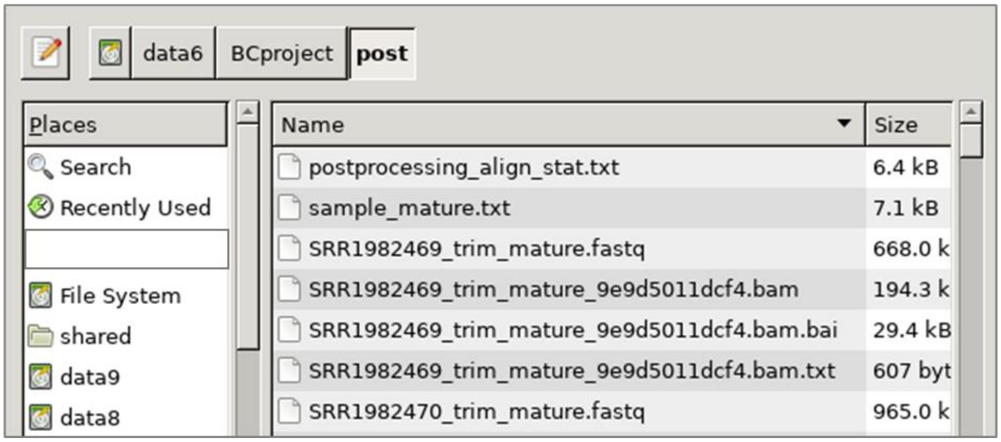

Figure 18. The output of postprocessing step

After counting of reads, the number of alignments in mature tRNAs is quantified and the result tables (tRNAs in rows and samples in columns) are produced for further analysis. There are three tables: individual, isodecoder, and isoacceptor levels of tRNAs (Figure 19).

The tables are saved in subdirectory ("$WORKDIR/rc") as below:

readcount_trnas.txt
readcount_isodecoders.txt
readcount_isoaccepters.txt

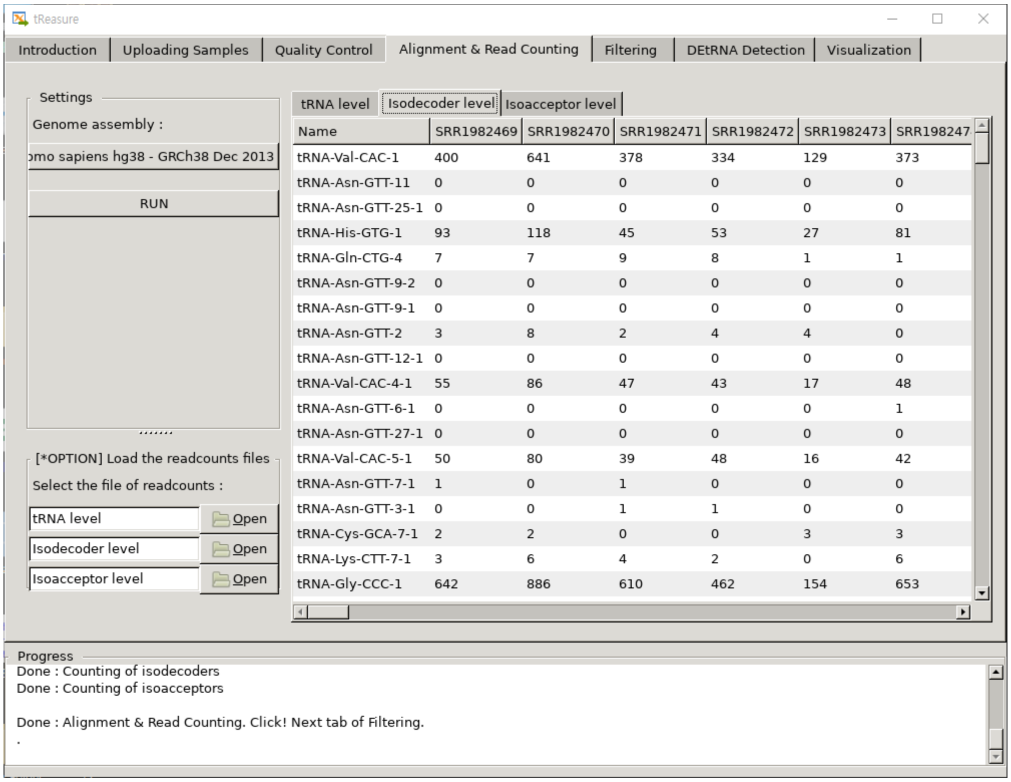

Figure 19. The output of read counting

Note. The process of status is displayed on the Bottom panel.

3.4. Tab “Filtering”

Before the statistical analysis, tReasure provides the function of filtering out the normalized genes having low read counts. The counts per gene were normalized to CPM (counts per million) using *cpm* function of *edgeR* packages[7].

3.4.1. Workflow of filtering

tReasure supports filtering out tRNA genes that does not have at least ‘m’ CPM value (0 to 10) in at least ‘n’ samples (0 to 100 %).

① Select value of Left panel (Figure 20).

② Click “RUN” button on the Left panel.

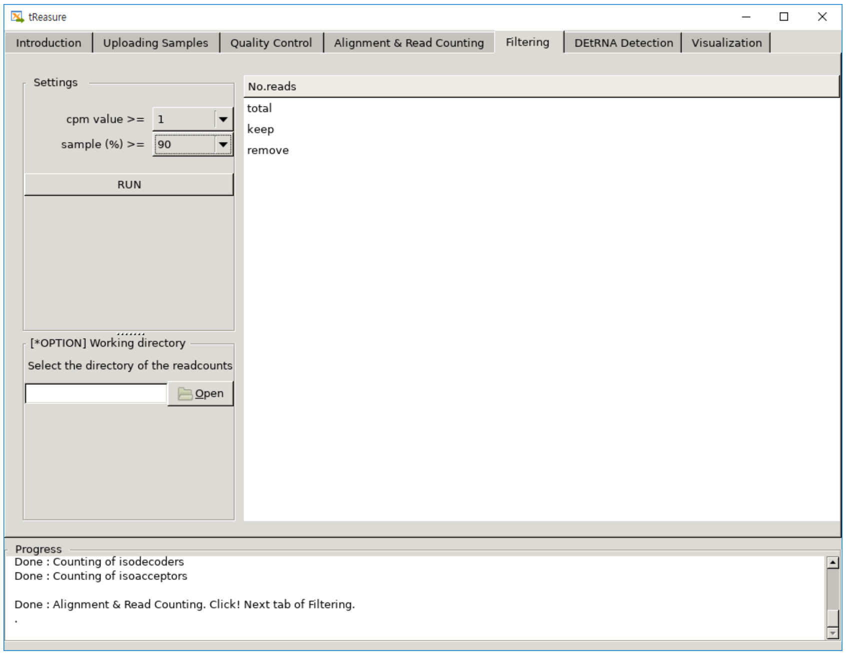

Figure 20. Tab of "**Filtering**"

⇨ The summary of filtering displays on the Right panel for "individual", "isodecoder", and "isoacceptor" levels of tRNAs (Figure 21). The tables save in subdirectory ("$WORKDIR/rc") as named files as below.

filtered_readcount_trnas.txt
filtered_readcount_isodecoders.txt
filtered_readcount_isoacceptors.txt

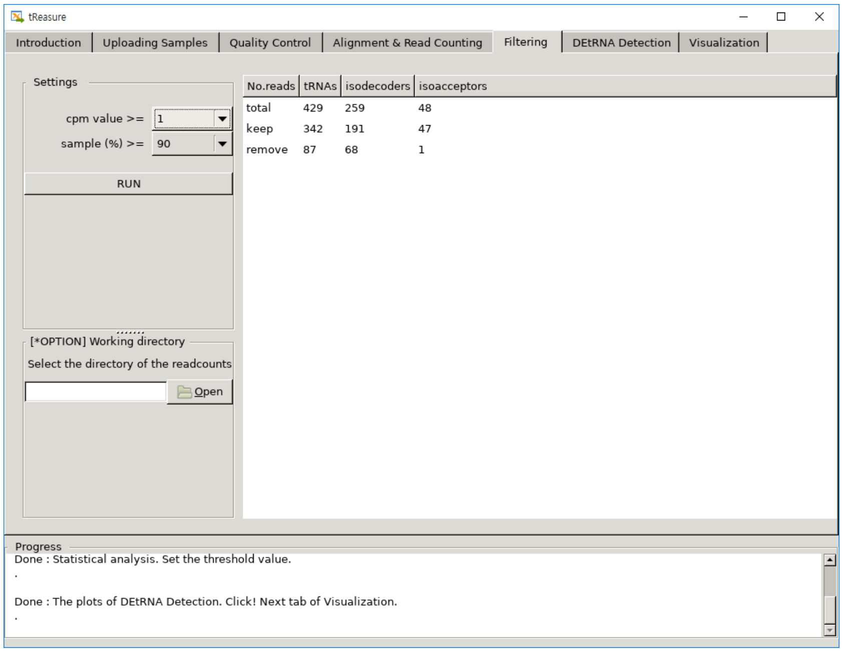

Figure 21. The summary of filtering

3.5. Tab "DEtRNA Detection"

For differentially expressed tRNA genes (DEtRNAs), tReasure provides three statistical methods: *DESeq2* [8], *EdgeR* [7] and *limma* [9]. They implement it in different methods for normalization. *DEseq2* use "Relative Log Expression" normalization (RLE), while *EdgeR* use "Trimmed Mean of *M*-value" normalization (TMM) method. *Limma* use "Quantile" normalization method.

In tReasure*, DEseq2* implements the statistical test using Walt test, while *EdgeR* implements likelihood ratio tests or quasi-likelihood F-tests as well as exact statistical methods for differential expression. *Limma* implements the empirical bayes statistics test method.

For multiple correction, tReasure provides three methods of FDR, Bonferroni correction, and Benjamini-Hochberg. For determining significance of different expression, adjusted p-value provides three value (0.001, 0.05 and 0.01) and the value of log2 fold-change from 0 to 2 increasing by 0.5.

3.5.1. Workflow of DEtRNA Detection

① Choose statistical method and set the statistical parameters.

As an example, quasi-likelihood F-test of *edgeR* was selected for statistical test (Figure 22).

② Click "RUN EdgeR" button of Left panel (Figure 22).

⇨ The results are display on Right panel and saved in subdirectory ($WORKDIR/stat/) named "stat_" as below. The data contains the value of logFC, logCPM, F-static, raw, and three adjusted p-value (FDR, Bonferroni, and Benjamini) for each tRNA (Figure 23).

stat_trna_list.txt
stat_isodecoder_list.txt
stat_isoacceptor_list.txt

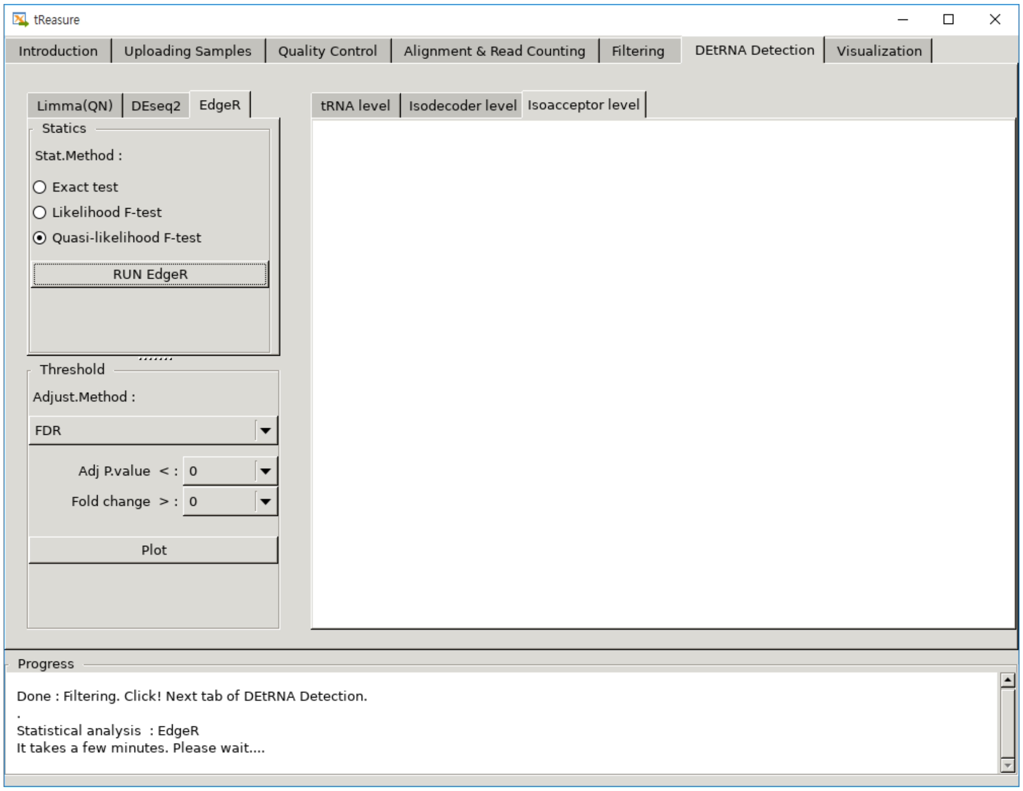

Figure 22. Tap of "**DEtRNA Detection**"

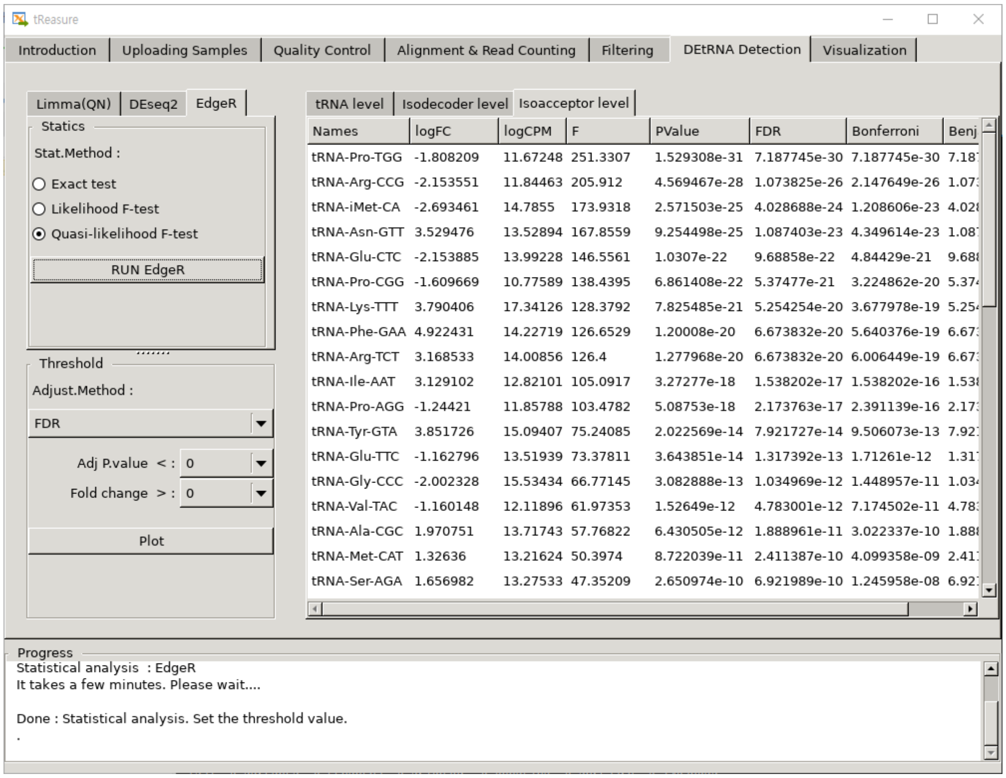

Figure 23. The output of “Run EdgeR”

③ Choose one adjustment method for multiple correction and set the threshold value in the Left panel (Figure 24) for detecting significant differential expression.

⇨ Filtered tRNAs are automatically reflected and showed on the Right panel (Figure 24). Also, filtered data is saved in subdirectory ($WORKDIR/stat/) named “DE” as below.

DEtrna_list.txt
DEisodecoder_list.txt
DEisoacceptor_list.txt

④ Click “Plot” button for visualization of tRNAs (Figure 24).

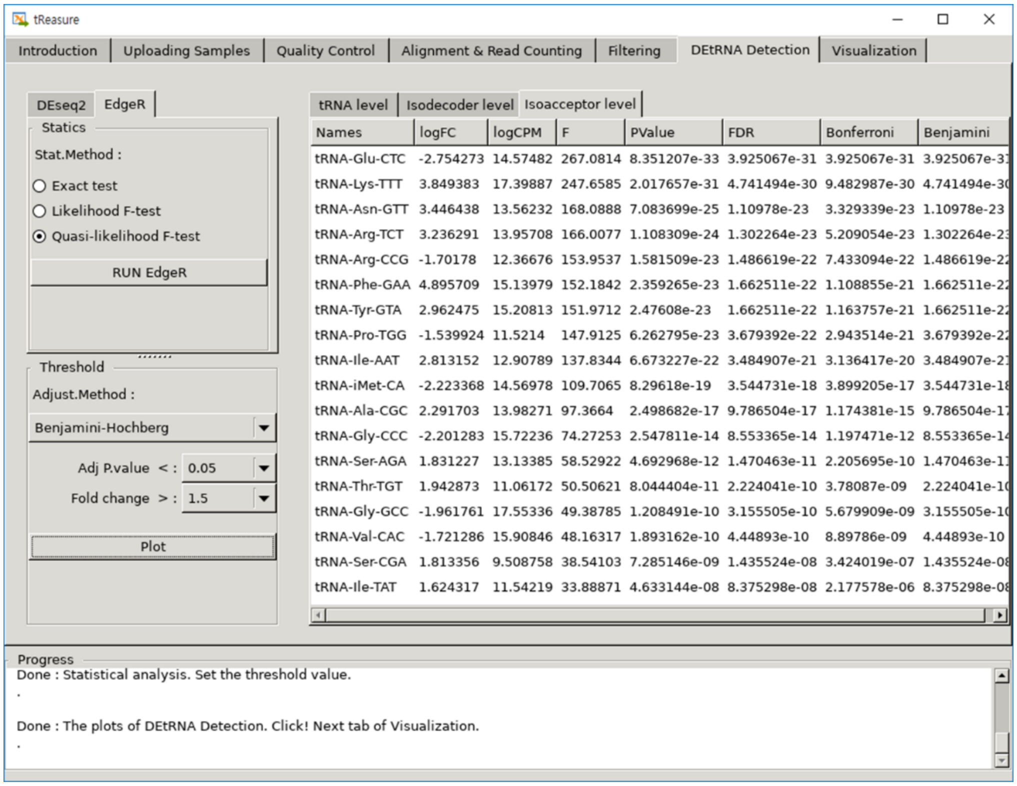

Figure 24. Statistical significance results

Note. The process of status is displayed on the Bottom panel.

3.6. Tab “Visualization”

For visualization, tReasure performed using *ggplot2* packages [10].

The plots generated in a previous step are displayed on windows and saved in the subdirectory (“$WORKDIR/stat/plot”) as a png format. Upon you clicking the tap of “Visualization”, there is no plot. In the Visualization tab, there are four sub-tabs: “MDS plot”, “Plot_trnas”, “Plot_isodecoders”, and “Plot_isoacceptors”. tReasure provide four kinds of plots for each tRNA levels as below.

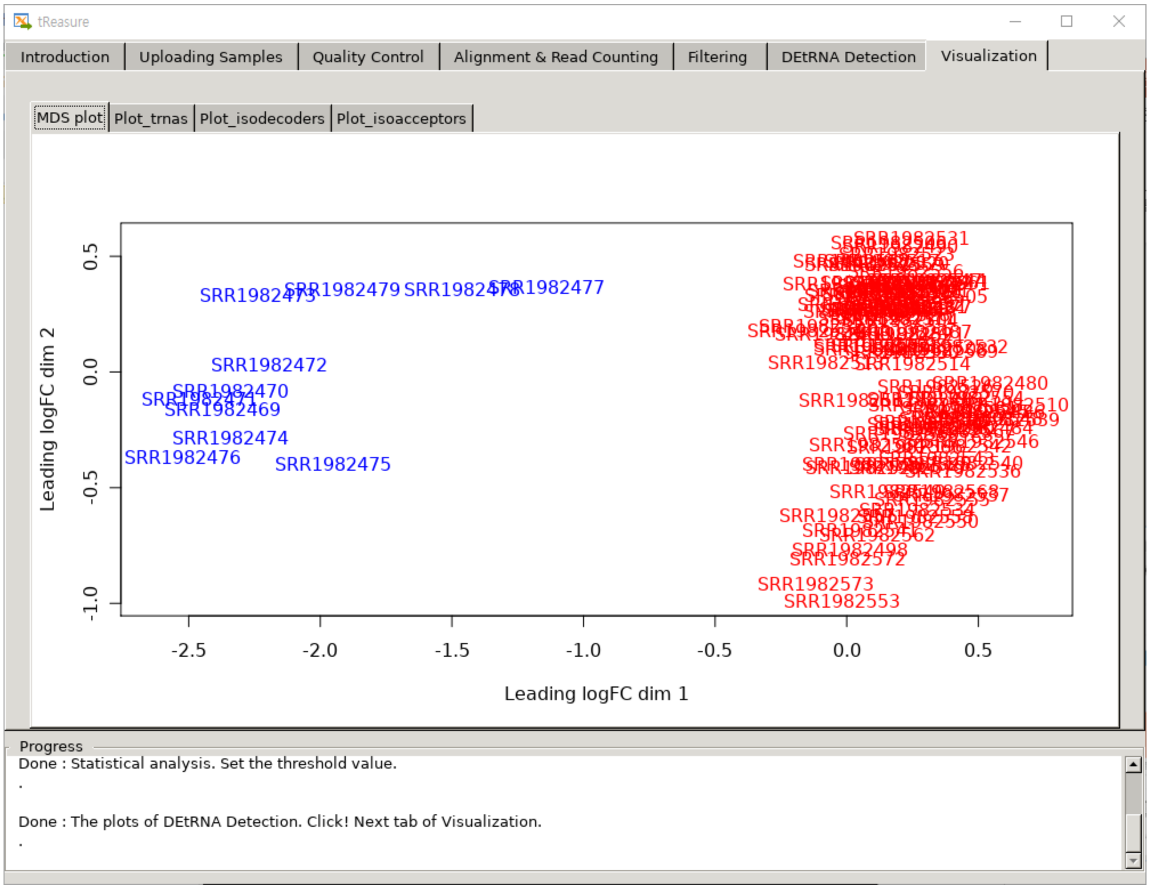

MDS plot shows the similarity/dissimilarity of the expression profiles between the samples (Figure 25).

Figure 25. MDS plot

Volcano plot shows statistical significance (adjusted p-value) versus magnitude of fold change using the analysis results of the “individual tRNA genes”.

For example, we identified 111 upregulated and 25 downregulated individual tRNAs in breast cancers compared with normal samples (Figure 26).

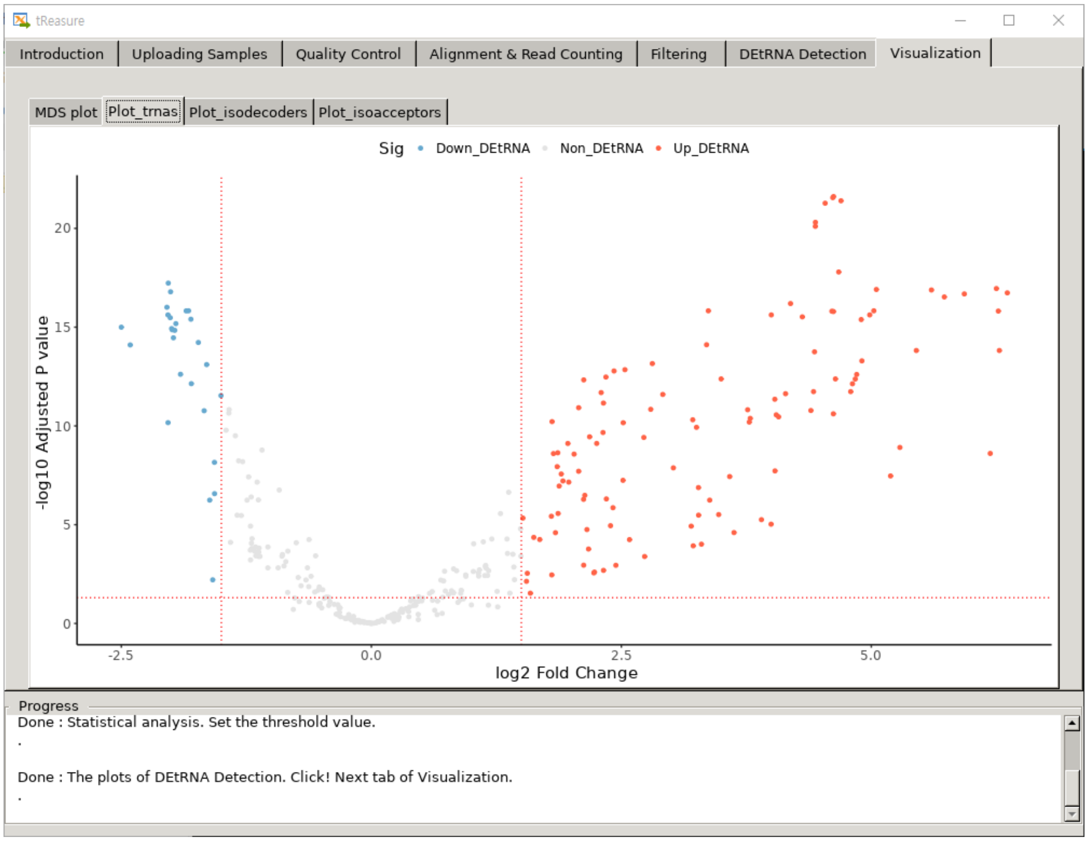

Figure 26. Volcano plot of individual tRNAs

Bar plot represents the frequency of significantly expressed tRNA-anticodons (adjusted p-value) using the results of the “isodecoders”.

For example, we identified 69 upregulated and 14 downregulated isodecoders in breast cancers compared with normal samples (Figure 27).

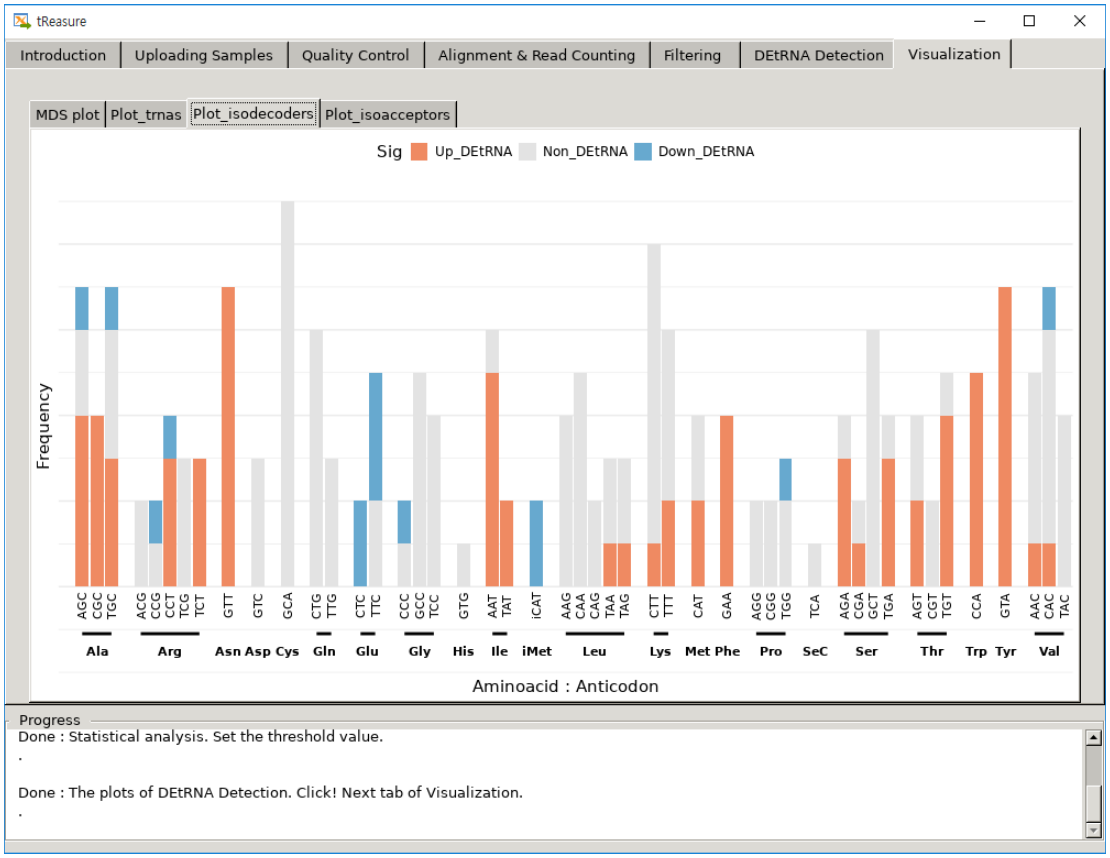

Figure 27. Bar plot for isodecoders

Pyramid plot displays the frequency of significantly expressed tRNA-amino acid using the results of the “isoacceptors”.

For example, we identified 10 upregulated and 7 downregulated isoacceptors in breast cancers compared with normal samples (Figure 28).

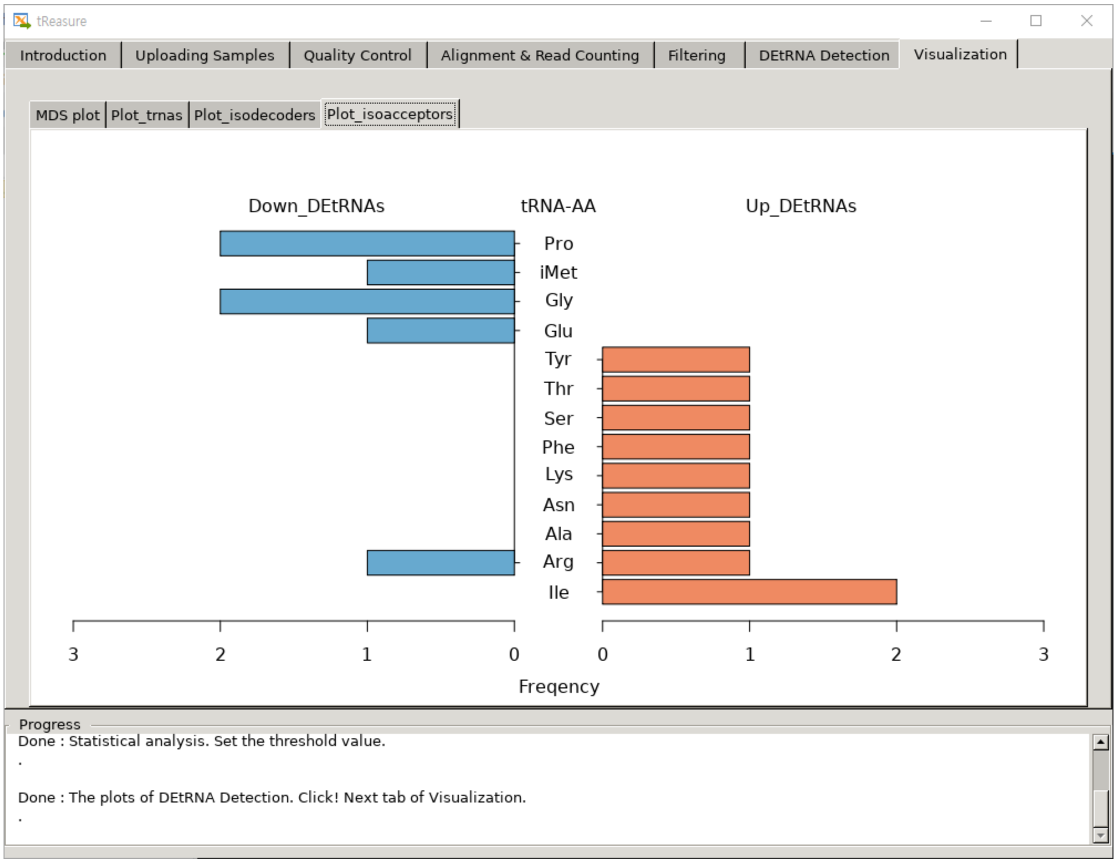

Figure 28. Pyramid plot for isoacceptors

3.6.1. Customizing plots

Users can modify plots (size, color, and so on) by loading files of plots, named “*.RData”, which are saved in the subdirectory (“$WORKDIR/stat/plot”). There are three RData files such as Volcano_Plot.RData, Pyramid_Plot.RData and Bar_Plot.RData. Each file contains one data. Frame. And one function objects to draw corresponding plot in different R session. You can modify the value in the code (Figure 29).

[Example: customizing a volcano plot]

① Open R or Rstudio

② Load package “ggplot2” and data “Volcano_Plot.RData”. Type on command window as below.

```
>library(ggplot2)
>load("$WORKDIR/sample/stat/plot/Volcano_Plot.RData")
>p()
>p()+theme_bw() # change theme
>p()+geom_point(size=3,aes(col=Sig)) # change dot size
>p()+scale_colour_manual(values = c(Non_DEtRNA="black", Up_DEtRNA="red",
 Down_DEtRNA="blue")) # change dot color
>ggsave("Volcanoplot.mod.png", p(), width = 10, height=10, dpi = 300,
 units = "in", limitsize=FALSE) # save a modified plot as "png"
```

③ Replace $WORKDIR depending on your environment (i.e., data6 /BCproject/stat/plot/Volcano_plot.Rdata).

⇨ You can find one data.frame object (“detRNA”), two values(“fc”, “pval”), and one function object of plot (“p”) on the Global Environment panel of R (or Rstudio). “detRNA” is a table of the statistical results and two values (“fc”, “pval”) are the pre-defined the threshold value.

④ Add (+) the value in “p()” plot object function. You can also resize and save the plots (Figure 29). For more customizing options, see details of ggplot2 options. https://www.rdocumentation.org/packages/ggplot2/versions/3.3.2

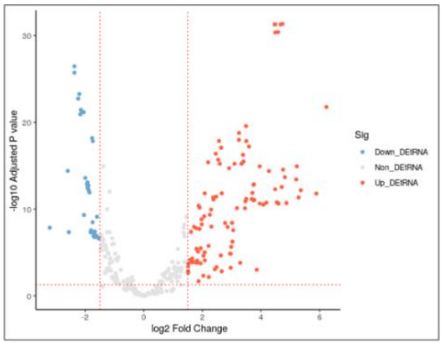

< Original >

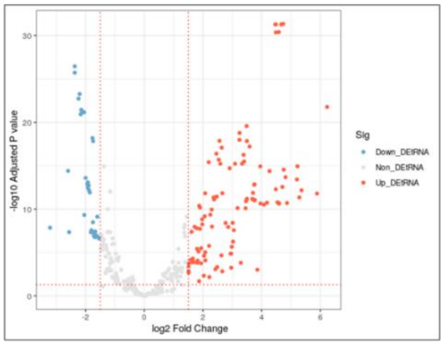

< Change theme>

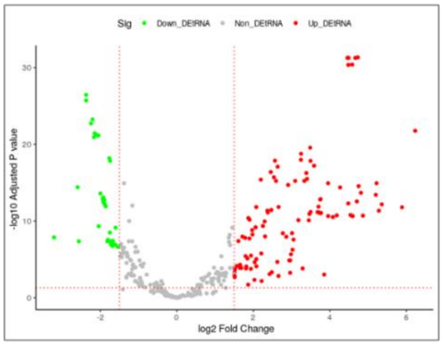

< Change dot color >

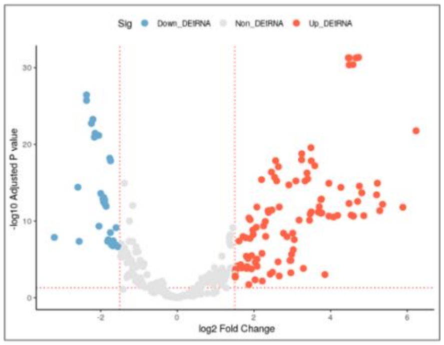

< Change dot size>

Figure 29. The outcome of customizing a volcano plot

[Example: customizing a pyramid plot]

① Open R or Rstudio

② Load package "Plotrix" and data "Pyramid_Plot.RData". Type on command window as below.

③ Replace $WORKDIR depending on your environment (i.e., data6 /BCproject/stat/plot/ Pyramid_Plot.RData).

⇨ You can find one data.frame object ("geneplot") and one function object of plot ("p") on the Global Environment panel of R (or Rstudio). You can find the "pyramid.plot(…)" in p() function.

```
>library(plotrix)
>load("./sample/stat/plot/Pyramid_Plot.RData")
>pyramid.plot(geneplot$Down_DEtRNA,
              geneplot$Up_DEtRNA,
              labels = >geneplot$Var1,
              lxcol = "#67A9CF", rxcol="#EF8A62", unit = "Freuency",
              gap=0.3, space = 0.15,
              top.labels = c("Down_DEtRNAs","tRNA-AA","Up_DEtRNAs"),
              laxlab= c(0,1,2,3),
              raxlab=c(0.1,2,3))
```

④ You can change the font size and save the plots.

⑤ Add the value (labelcex= 1.3) in "pyramid.plot(..)"

```
>library(plotrix)
>load("./sample/stat/plot/Pyramid_Plot.RData")
>pyramid.plot(geneplot$Down_DEtRNA,
              geneplot$Up_DEtRNA,
              labels = >geneplot$Var1,
              lxcol = "#67A9CF", rxcol="#EF8A62", unit = "Freuency",
              gap=0.3, space = 0.15,
              top.labels = c("Down_DEtRNAs","tRNA-AA","Up_DEtRNAs"),
              laxlab= c(0,1,2,3),
              raxlab=c(0.1,2,3),
              labelcex = 1.3) #add the font value
```

4. Option

➢ **Case 1.** If user wants to re-analyze with new statistical analysis parameters,

Re-analyze with new statistical analysis parameters should go back the step after "**Alignment and Read counting**" tab.

① Click "**Filtering**" tab. Bottom sided of Left panel have option section.

② Set the previous or the folder that you want to reanalyze with new statistical analysis.

Note. The working directory should contain raw FASTQs (e.g., /data6/BCproject). Before doing statistical analysis, you could check the table of read counts by loading files on the Left panel of **"Alignment and Read counting "**tab (Figure18).

- **Case 2.** If user finish the read counting and couldn't finish filtering,

  In case user exit tReasure before filtering, user needs to reset the working directory on the Left panel of "**Filtering**" tab (Figure 20).

  ① Click **"Filtering"** tab.

  ② Reset the working directory on the bottom sided of Left panel.

  ③ Start analyzing filtering (page 17).

5. Reference

1. Krishnan P, Ghosh S, Wang B, Heyns M, Li D, Mackey JR, Kovalchuk O, Damaraju S: **Genome-wide profiling of transfer RNAs and their role as novel prognostic markers for breast cancer**. *Sci Rep* 2016, **6**:32843.
2. Gaidatzis D, Lerch A, Hahne F, Stadler MB: **QuasR: quantification and annotation of short reads in R**. *Bioinformatics* 2015, **31**(7):1130-1132.
3. Morgan M PH, Obenchain V, Hayden N: **Rsamtools: Binary alignment (BAM), FASTA, variant call (BCF), and tabix file import**. *http://bioconductororg/packages/Rsamtools* 2021.
4. Hoffmann A, Fallmann J, Vilardo E, Morl M, Stadler PF, Amman F: **Accurate mapping of tRNA reads**. *Bioinformatics* 2018, **34**(7):1116-1124.
5. Chan PP, Lowe TM: **GtRNAdb 2.0: an expanded database of transfer RNA genes identified in complete and draft genomes**. *Nucleic Acids Res* 2016, **44**(D1):D184-189.
6. Torres AG, Reina O, Stephan-Otto Attolini C, Ribas de Pouplana L: **Differential expression of human tRNA genes drives the abundance of tRNA-derived fragments**. *Proc Natl Acad Sci U S A* 2019, **116**(17):8451-8456.
7. Robinson MD, McCarthy DJ, Smyth GK: **edgeR: a Bioconductor package for differential expression analysis of digital gene expression data**. *Bioinformatics* 2010, **26**(1):139-140.
8. Love MI, Huber W, Anders S: **Moderated estimation of fold change and dispersion for RNA-seq data with DESeq2**. *Genome Biol* 2014, **15**(12):550.
9. Ritchie ME, Phipson B, Wu D, Hu Y, Law CW, Shi W, Smyth GK: **limma powers differential expression analyses for RNA-sequencing and microarray studies**. *Nucleic Acids Res* 2015, **43**(7):e47.
10. Wickham H: **ggplot2: Elegant Graphics for Data Analysis.** . *Springer-Verlag New York* 2016.
